# LPS resistance of SPRET/Ei mice is mediated by Gilz, encoded by the *Tsc22d3* gene on the X chromosome

**DOI:** 10.1002/emmm.201201683

**Published:** 2013-03-05

**Authors:** Iris Pinheiro, Lien Dejager, Ioanna Petta, Sofie Vandevyver, Leen Puimège, Tina Mahieu, Marlies Ballegeer, Filip Van Hauwermeiren, Carlo Riccardi, Marnik Vuylsteke, Claude Libert

**Affiliations:** 1 Department for Molecular Biomedical ResearchVIB, Ghent, Belgium; 2 Department of Biomedical Molecular Biology, Ghent UniversityGhent, Belgium; 3 Department of Plant Systems Biology, VIBGhent, Belgium; 4 Department of Plant Biotechnology and Bioinformatics, Ghent UniversityGhent, Belgium; 5 Department of Clinical and Experimental Medicine, Section of Pharmacology, University of PerugiaPerugia, Italy

**Keywords:** Gilz, inflammation, LPS, SPRET/Ei, X chromosome

## Abstract

Natural variation for LPS-induced lethal inflammation in mice is useful for identifying new genes that regulate sepsis, which could form the basis for novel therapies for systemic inflammation in humans. Here we report that LPS resistance of the inbred mouse strain SPRET/Ei, previously reported to depend on the glucocorticoid receptor (GR), maps to the distal region of the X-chromosome. The GR-inducible gene *Tsc22d3*, encoding the protein Gilz and located in the critical region on the X-chromosome, showed a higher expressed SPRET/Ei allele, regulated in *cis*. Higher Gilz levels were causally related to reduced inflammation, as shown with knockdown and overexpression studies in macrophages. Transient overexpression of Gilz by hydrodynamic plasmid injection confirmed that Gilz protects mice against endotoxemia Our data strongly suggest that Gilz is responsible for the LPS resistance of SPRET/Ei mice and that it could become a treatment option for sepsis.

## INTRODUCTION

Sepsis, a common systemic inflammatory reaction to microbial infections, imposes a huge social and economic burden on healthcare systems and remains associated with very high mortality rates (40–60%) (Vincent et al, 2006). Despite decades of intensive research and numerous clinical trials, only few new therapies are relatively beneficial, and current management of sepsis relies mainly on supportive treatments (Riedemann et al, 2003). Thus, there is an urgent need for new therapeutic targets for the treatment of this condition.

Several mouse models of sepsis have been developed, ranging from exogenous administration of pathogens or toxins, such as lipopolysaccharide (LPS), to surgical procedures such as cecal ligation and puncture (CLP) (Dejager et al, 2011). Injection of LPS, a component of the outer leaflet of the outer membrane of Gram-negative bacteria, reproducibly mimics many of the pathophysiologic manifestations of sepsis (Schultz & van der Poll, 2002).

We have been studying the mouse strain SPRET/Ei, derived from *Mus spretus*, to find new molecular players in LPS-induced inflammation. Among their many different phenotypes (Dejager et al, 2009), SPRET/Ei (S) mice exhibit a remarkable resistance to LPS (Dejager et al, 2010b; Mahieu et al, 2006) and to Gram-negative infections (Dejager et al, 2010a) compared to the commonly used laboratory mouse strain, C57BL/6 (B). These phenotypes make this strain useful for identifying new molecular targets for the treatment of sepsis. In addition, because *Mus spretus* had diverged from *Mus musculus* at least 1.5 million years ago (Guenet & Bonhomme, 2003), SPRET/Ei mice possess a larger degree of genetic polymorphisms than the common laboratory strains. This makes them very useful in linkage studies searching for the genetic variants responsible for the interesting characteristics of this mouse strain (Dejager et al, 2009).

We previously reported the resistance of SPRET/Ei mice to LPS as a dominant trait (Mahieu et al, 2006) and its strong dependence on the anti–inflammatory actions of the glucocorticoid receptor (GR) (Dejager et al, 2010b). Glucocorticoids (GCs) are potent anti-inflammatory agents frequently used to treat inflammatory diseases, such as asthma (Rhen & Cidlowski, 2005). However, the beneficial effect of GCs in sepsis remains controversial, so GCs are mainly used to limit the use of vasopressors and to counterbalance adrenal insufficiency. By binding to the GR, GCs modulate the immune response by transrepression (TR) of key inflammatory transcription factors, such as NFκB, and by transactivation (TA) of anti-inflammatory genes containing glucocorticoid responsive elements (GRE) (Ayroldi & Riccardi, 2009). For example, the prominent role of the GC-induced GRE-gene *Dusp1* (encoding dual specific phosphatase 1) in the control of excessive inflammation has been confirmed in Dusp1 deficient mice, which are very sensitive for LPS and TNF-induced lethal inflammation (Vandevyver et al, 2012; Zhao et al, 2005) and in other sepsis models (Frazier et al, 2009; Hammer et al, 2010). Another important anti-inflammatory GRE gene is *Tsc22d3* (TSC22 domain family member 3), also known as Gilz (glucocorticoid-induced leucine zipper) (D'Adamio et al, 1997). Gilz mimics many of the anti-inflammatory actions of the GR *in vitro* (Ayroldi & Riccardi, 2009; Beaulieu & Morand, 2011). The anti-inflammatory actions of Gilz were recently confirmed in several inflammatory diseases, *i.e.* inflammatory bowel disease (Cannarile et al, 2009), rheumatoid arthritis (Beaulieu et al, 2010) and multiple sclerosis (Srinivasan & Janardhanam, 2011), but the role of Gilz in endotoxemia and sepsis is unknown.

The objective of this study was to map the loci and to identify the gene(s) underlying the LPS resistance of SPRET/Ei mice, using linkage analysis of 140 backcross mice derived from a cross between (BxS)F1 (♀) and C57BL/6 (♂) (BSB backcross), followed by a candidate gene approach. We identified two quantitative trait loci (QTL), of which one mapped on the X chromosome (60-70cM). We hypothesized that *Tsc22d3,* encoding the protein Gilz, underlies the X-located QTL because the LPS resistance of SPRET/Ei mice depends on GR activity (Dejager et al, 2010b) and its anti-inflammatory properties. Expression and functional studies, in macrophages as well as *in vivo*, suggested Gilz as the primary candidate for explaining the LPS-resistance in SPRET/Ei mice and as an important novel protective factor in endotoxemia.

## RESULTS

### The extreme LPS resistance of SPRET/Ei mice maps to the distal region of the X chromosome

To identify the loci responsible for the LPS resistance of SPRET/Ei mice, we analyzed a backcross population of 140 mice obtained by crossing (BxS)F1 hybrid females with C57BL/6 males. We injected the parental lines, the (BxS)F1 hybrid and the 140 offspring with 250 µg of LPS and followed mortality for five days. Both the parental SPRET/Ei mice and the (BxS)F1 hybrid showed increased LPS resistance, which is consistent with the dominant inheritance of SPRET/Ei LPS resistance previously reported (Dejager et al, 2010b; Mahieu et al, 2006).

**Figure 1:**
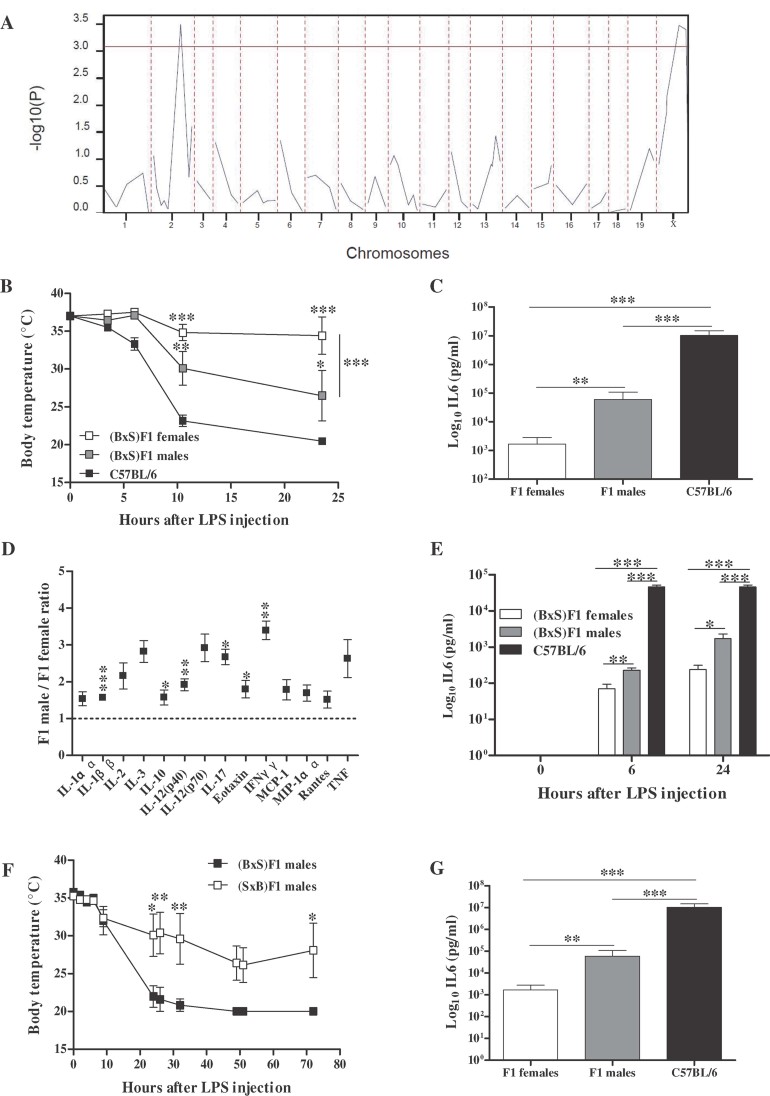
SPRET/Ei LPS resistance is genetically linked to the distal region of the X chromosome In graphs **(B-G)** data are expressed as mean ± s.e. and significances were calculated using Mann Whitney test. *, ** and *** represent *P* < 0.05, *P* < 0.01 and *P* < 0.001, respectively. **A.** QTL mapping of survival after LPS challenge in 140 backcross mice. Linkage scores (plotted as –log_10_(*P*)) are shown according to genome position. The linkage analysis indicates that two loci, on chromosomes 2 and X, contain a QTL conferring resistance to LPS in SPRET/Ei mice. On chromosome 2, the highest score maps to D2Mit510 (–log_10_(*P*) = 3.49, Wald test), a marker located at 65 cM (118 Mb). In addition, the distal portion of the X chromosome, and specifically the marker DXMit135 (–log_10_(*P*) = 3.4, Wald test) located at 69 cM (161 Mb), was significantly linked to this trait.**B.** (BxS)F1 females are more resistant to LPS than their male counterparts. Female (BxS)F1 (n = 6), male (BxS)F1 (n = 6) and C57BL/6 (n = 6) mice were injected i.p. with 500 µg of LPS and body temperature was monitored. This experiment was repeated four times and we obtained similar trends. Data from one representative experiment are shown here.**C.** Serum IL6 levels after LPS injection in (BxS)F1 mice are lower in females. Blood was obtained from female (BxS)F1 (n = 10), male (BxS)F1 (n = 10) and C57BL/6 (n = 10) mice 6 h after i.p. injection with 500 µg of LPS. The data in graph C were repeated by three independent experiments.**D.** After LPS injection, (BxS)F1 females produce lower levels of pro-inflammatory cytokines and chemokines than corresponding males. (BxS)F1 females (n = 5) and males (n = 5) were injected i.p. with 500 µg of LPS and blood was collected 6 h later. The male/female ratio of the mean values was calculated. Ratios > 1.0 indicate higher levels of cytokines and chemokines in F1 males. The levels of these cytokines were measured once.**E.** LPS-stimulated bone marrow derived macrophages (BMDM) (n = 6) derived from (BxS)F1 females produce significantly less IL6 than BMDM from (BxS)F1 males and C57BL/6 mice. This experiment was performed at least 3 times; similar trends were obtained each time.**F–G.** (SxB)F1 males are more resistant to LPS than (BxS)F1 males. (SxB)F1 males (n = 4) and (BxS)F1 males (n = 4) were injected *i.p.* with 500 µg of LPS and body temperature was monitored. **(F)**. Blood was collected 6 h after LPS challenge and serum IL6 levels were measured **(G)**. The results indicated in graphs **(F)** and **(G)** were derived from 1 experiment due to a lack of (SxB)F1 male mice.

All 140 offspring, of which 90 (64%) survived the LPS challenge, were genotyped using 87 microsatellite markers evenly distributed over the genome, followed by a genome-wide linkage analysis. The genome-wide significance threshold for detection of QTL co-segregating with LPS resistance was set at *P* = 0.05, corresponding with a threshold of (–log_10_(*P*) = 3.086) (Li & Ji, 2005). Two genome-wide significant QTL were identified, one on chromosome 2 (marker D2Mit510 at 65 cM; –log_10_(*P*) = 3.49, Wald test) and one on the X chromosome (marker DXMit135 at 69 cM; –log_10_(*P*) = 3.4, Wald test) (Fig 1A), spanning a 10cM interval. Linkage analysis performed for both sexes separately resulted in identical QTL, though, at lower significance (Supporting Information Fig. S1B). At both QTL, the presence of a SPRET/Ei allele inferred an increase in LPS resistance. Both QTL did not exhibit epistasis, suggesting that each QTL contributed additively to the survival rates (Supporting Information Fig. S1A).

When (BxS)F1 hybrid mice were injected with different doses of LPS, the females (carrying a SPRET/Ei X chromosome) showed better survival (500 µg LPS, *p* = 0.03 and 750 µg LPS, *p* = 0.05) than the males (carrying the C57BL/6 X chromosome). This confirms the strong involvement of the X chromosome in the LPS resistance of SPRET/Ei mice and the dominant mode of inheritance of this trait. (BxS)F1 females also suffered less from LPS-induced hypothermia (Fig 1B) and produced less IL6 (*p* = 0.0014) (Fig. 1C) and other cytokines and chemokines (Fig 1D) than (BxS)F1 males, which indicates that they are less sensitive to acute endotoxemia. In addition, the increased LPS resistance of the (BxS)F1 females was also reflected at the level of macrophages as LPS-stimulated bone-marrow derived macrophage cultures (BMDM) derived from female (BxS)F1 mice displayed significantly less IL6 production compared to BMDM from (BxS)F1 males (*p* = 0.0043), which, in turn, had less cytokine production compared to BMDM from C57BL/6 mice (*p* = 0.0008) (Fig 1E). In conclusion, (BxS)F1 females are more resistant to endotoxemia than (BxS)F1 males, which is compatible with a protective role of the SPRET/Ei X chromosome against endotoxemia.

(SxB)F1 and (BxS)F1 males are genetically identical except for their X and Y chromosomes and some imprinted genes. When these F1 mice were injected with 500 µg of LPS, the (SxB)F1 mice (carrying a SPRET/Ei X chromosome) displayed better survival (*p* = 0.027), smaller drop in body temperature (Fig 1F) and lower production of IL6 production (*p* = 0.0014) (Fig 1G) than the (BxS)F1 mice. These results confirm again the involvement of the SPRET/Ei X chromosome in the response to LPS.

To exclude the involvement of sex hormones in LPS resistance, we first compared the LPS resistance in ovariectomized (ovx) (BxS)F1 mice with their sham operated (BxS)F1 counterparts. After injection with 500 µg of LPS, all ovx and sham operated mice survived, showing no significant differences in body temperature and IL6 levels (*p* = 0.9781) (Supporting Information Fig. S2A and B; IL6). A similar experiment was performed on males to examine the possible role of testosterone in sensitizing (BxS)F1 males for LPS by injecting castrated (orchiectomy) and sham operated mice with 500 µg of LPS. Mortality, body temperature (Supporting Information Fig. S2C) and serum IL6 levels (Supporting Information Fig S2D; *p* = 0.5228) were not significantly different between the two groups. These findings exclude a role of sex hormones in the higher LPS resistance of (BxS)F1 females relative to (BxS)F1 males.

### Identification of *Tsc22d3* as a candidate gene

**Figure 2:**
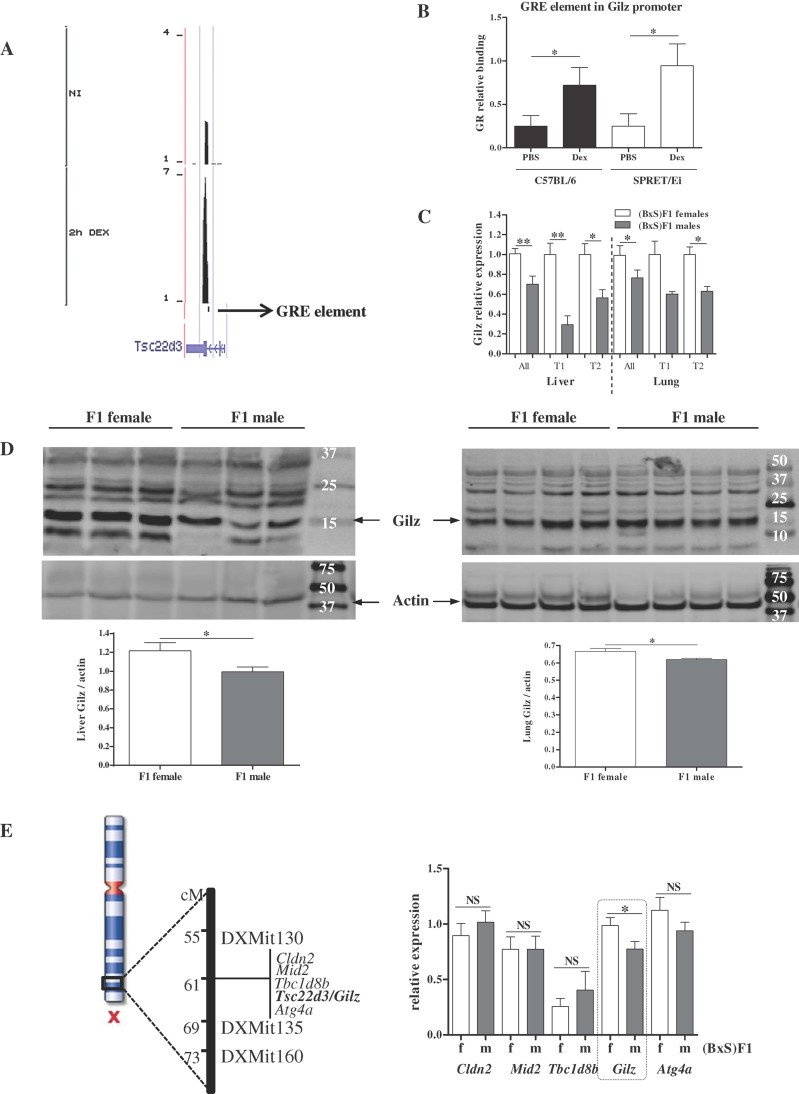
Gilz is the candidate LPS resistance gene on the X chromosome In graphs **(B-E)**, data are expressed as mean ± s.e. and significances were calculated using Mann Whitney test. *, ** and *** represent *P* < 0.05, *P* < 0.01 and *P* < 0.001, respectively. ChIP-Seq analysis shows GR-DNA binding at GRE element in the *Tsc22d3/Gilz* locus. ChIP-seq experiments were performed on hepatoma BWTG3 cells that either were not treated (NI) or were treated for 2 h with 10^−6^ M Dexamethasone (Dex), a synthetic GR ligand. WIG data files were uploaded in the UCSC genome browser to visualize GR binding locations (depicted as black peaks) in both conditions. In addition, the GRE sequence, identified using MEME motif analysis, was uploaded to locate GRE sequences in the genome, depicted as a black line. Here the GRE in the promoter of the gene *Tsc22d3*, on which liganded GR binds, is shown.GR binds to the GRE element in the promoter of Gilz in livers of C57BL/6 and SPRET/Ei mice. ChIP experiments were performed on liver lysates from C57BL/6 and SPRET/Ei mice stimulated with PBS (n = 4) or with 200 µg Dex (2 h; n = 3). GR–DNA binding at a GRE located in the promoter of Gilz is shown here; the results are obtained from two independent experiments.(BxS)F1 females exhibit higher levels of *Gilz* mRNA than (BxS)F1 males. Basal liver (left) and lung (right) samples were collected for RNA preparation from unstimulated female and male (BxS)F1 mice. Gilz expression levels were measured with QPCR using primers, which recognize all transcript variants (all, n = 27, combined data from three independent experiments), the canonical isoform (T1, n = 4, one experiment) or the smallest isoform (T2, n = 4, one experiment). Expression levels are expressed relative to the mean of the female F1 expression levels.(BxS)F1 females exhibit higher levels of Gilz protein than (BxS)F1 males. Basal liver (left graph) and lung (right graph) samples were collected from female (n = 8) and male (n = 8) (BxS)F1 mice. Western blots for Gilz (15 kDa) and β-actin (42 kDa) are shown above (a representative gel containing some of the samples is shown), and the normalized Gilz protein levels are depicted below. These results were repeated in at least three different experiments. Source data is available for this figure in the Supporting Information.Higher Gilz expression levels in (BxS)F1 females are not a consequence of escape from X inactivation. mRNA levels of different closely located genes on the X chromosome (61 cM, left picture) were measured in livers of unstimulated female (BxS)F1 (n = 5) and male (BxS)F1 (n = 6) mice. This experiment was performed twice and results from one representative experiment are shown.

We previously described the important role of the GR in mediating the LPS resistance of SPRET/Ei mice (Dejager et al, 2010b). Therefore, we focused on 41 putative *in silico* predicted GRE-genes located in a ∼10 cM region flanking the X-located QTL peak (Supporting Information Table 1). Next, ChIP-Seq analysis, studying the binding of GR to DNA upon dexamethasone (Dex) stimulation of mouse hepatoma cells, identified *Tsc22d3* as the only GC-inducible gene in this QTL region (Fig 2A). Additionally, ChIP analysis of liver lysates confirmed that GR binds significantly to a GRE element in the promoter of *Tsc22d3* of C57BL/6 and SPRET/Ei mice (Fig 2B; *p* = 0.0442 and *p* = 0.0492, respectively). Given the considerable anti-inflammatory potential of Gilz (Ayroldi et al, 2001; Mittelstadt & Ashwell, 2001), we hypothesized that *Tsc22d3* is the causative gene underlying the X-located QTL for LPS resistance. Previously, we reported higher expression levels of Gilz in livers of SPRET/Ei mice compared to C57BL/6 mice (Dejager et al, 2010b). Here, we investigated Gilz mRNA and protein levels in unstimulated samples of liver and lung from (BxS)F1 mice and found that the levels of Gilz mRNA, total (*p* = 0.0007 in liver samples; *p* = 0.0012 in lung samples) as well as of the different isoforms (*p* = 0.0028 and *p* = 0.0176 in the liver samples), were significantly higher in females than in males (Fig 2C). The protein levels reflected the Gilz expression levels (Fig 2D; *p* = 0.0352 in liver and *p* = 0.0410 in lung). Expression analysis of the genes located on the X chromosome close to the *Tsc22d3* gene showed that only the levels of Gilz were significantly increased in (BxS)F1 females compared to (BxS)F1 males (*p* = 0.0296) (Fig 2E), ensuring that X inactivation escape was not the cause of the increased Gilz expression levels in (BxS)F1 females.

### Increased Gilz levels in female (BxS)F1 mice are associated with a sequence variation in the *Tsc22d3* locus on chromosome X

**Figure 3:**
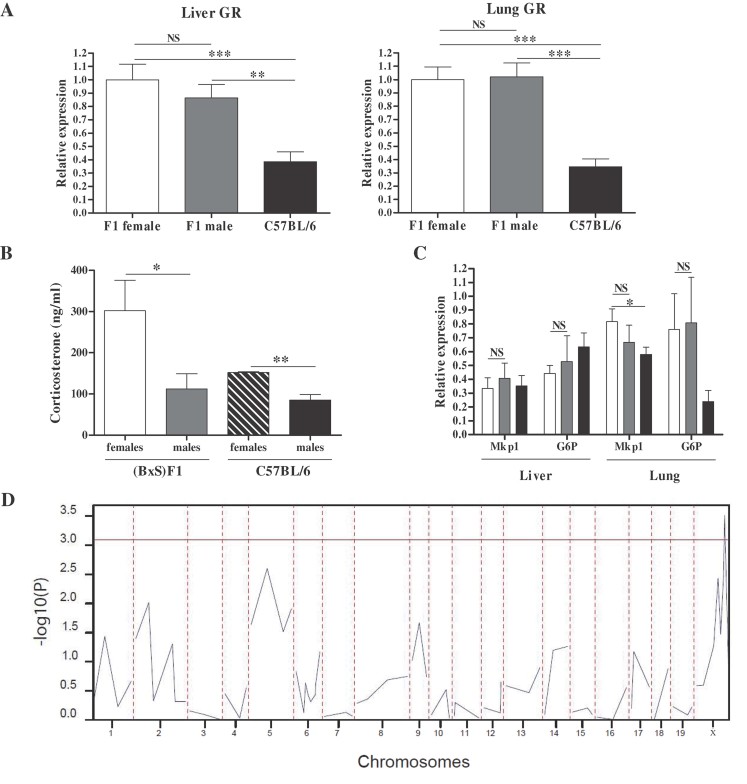
High Gilz levels in F1 females are not mediated by increased GR levels or activity The results from graphs **(A-C)** were repeated in at least three different experiments. In graphs **(A-D)**, data are expressed as mean ± s.e. and significances were calculated using Mann Whitney test. *, ** and *** represent *P* < 0.05, *P* < 0.01 and *P* < 0.001, respectively. (BxS)F1 females and males have equal levels of *GR* mRNA, but both females and males have higher levels than C57BL/6 mice. Liver (left graph) and lung (right graph) samples were collected for RNA preparation from female (BxS)F1 (n = 27), male (BxS)F1 (n = 27) and C57BL/6 (n = 18) mice, collected from three independent experiments; expression levels are expressed relative to the mean of the female (BxS)F1 expression levels.Female mice show increased levels of corticosterone. Corticosterone levels were measured in circulation of (BxS)F1 (n = 8) and C57BL/6 mice (n = 5) of both sexes.GR-transactivated genes such as *Mkp1* and *G6P* are not differentially expressed in (BxS)F1 females and males. Liver and lung samples were collected for RNA preparation from unstimulated female (BxS)F1 (white bars, n = 8), male (BxS)F1 (grey bars, n = 8) and C57BL/6 (black bars, n = 8) mice.High levels of Gilz mRNA in SPRET/Ei mice are genetically linked to the *Tsc22d3* (*Gilz*) locus on chromosome X. Linkage interval mapping using the GenStat program revealed that a locus on chromosome X is strongly linked to the increased *Gilz* expression levels. The genome-wide significance threshold for detection of QTL co-segregating with variation in Gilz expression was set at *P* = 0.05, corresponding to a threshold of –log_10_(*P*) = 3.086, depicted as the red line in the graph. Only one genome-wide significant QTL was identified on chromosome X (marker DXMit34,–log_10_(P) = 3.51, Wald test,), at 2 cM from the Gilz (marker Gilz, –log_10_(P) = 2.47, Wald test).

We previously reported that the higher levels of Gilz mRNA in SPRET/Ei mice compared to C57BL/6 mice can be partly attributed to a sequence variant in the GR gene, resulting in increased GR activity and levels (Dejager et al, 2010b). In this study, GR expression levels were significantly higher in (BxS)F1 mice than in C57BL/6 mice (Fig 3A) in unstimulated livers (*p* = 0.0003 and *p* = 0.0014, female and male F1 mice, respectively) and lungs (*p* < 0.0001 in both groups), but did not differ between male and female (BxS)F1 mice, illustrating that GR levels do not account for the higher Gilz levels in (BxS)F1 females. In addition, corticosterone (CS) levels were significantly different (*p* = 0.021) between (BxS)F1 females and males (Fig 3B). Among rodents, females have generally higher CS levels than males. A similar pattern was identified for C57BL/6 female mice compared to C57BL/6 males (*p* = 0.0032) (Fig 3B). However, this means that the higher *Gilz* transcript levels in (BxS)F1 females might have been due to higher CS levels and thus GR activity, rather than higher GR levels. To further test dependency of higher Gilz levels in (BxS)F1 females on GR activity, we determined the expression levels of other GRE-genes known to be induced by CS-bound GR, *Mkp1* and *G6P* (glucose 6 phosphatase). (BxS)F1 females and males displayed equal GR activity, as shown by similar expression levels of GRE-genes in unstimulated livers and lungs (Fig 3C). These data suggest that the difference in Gilz mRNA levels between (BxS)F1 females and males is independent of GR expression levels and activity.

Linkage analysis of liver Gilz mRNA levels measured in 172 offspring, derived from a backcross between (BxS)F1 females and C57BL/6 males, demonstrated a QTL strongly linked to marker DXMit34 located at 63 cM on the chromosome X (–log_10_(*P*) = 3.51, Wald test). Next, a marker located in the *Tsc22d3* gene and polymorphic between SPRET/Ei and C57BL/6 was added and it showed genetic linkage to Gilz mRNA levels (–log_10_(*P*) = 2.47, Wald test). Note that the limited number of backcross mice that were genotyped for this marker likely accounts for the reduced linkage (Fig 3D). The SPRET/Ei allele inferred an increase in Gilz expression at this QTL. The local QTL at *Tsc22d3* very likely indicates the presence of *cis*-acting variant(s) contributing to the higher expression of Gilz in SPRET/Ei compared to C57BL/6. A comparison of the full-length sequence of the *Tsc22d3* locus in *Mus musculus* and *Mus spretus* showed five synonymous sequence variations and one missense substitution (107E > Q) at the coding level. This position is located in the PER region (proline and glutamic acid rich domain), which is essential for Gilz-mediated NFκB repression (Di Marco et al, 2007). It affects the three different protein-coding isoforms of Gilz, because they share the same C-terminal (D'Adamio et al, 1997). In addition, several other sequence variants were found in upstream and downstream regulatory regions (Supporting Information Fig S3).

### Increased Gilz levels protect against endotoxemia

**Figure 4:**
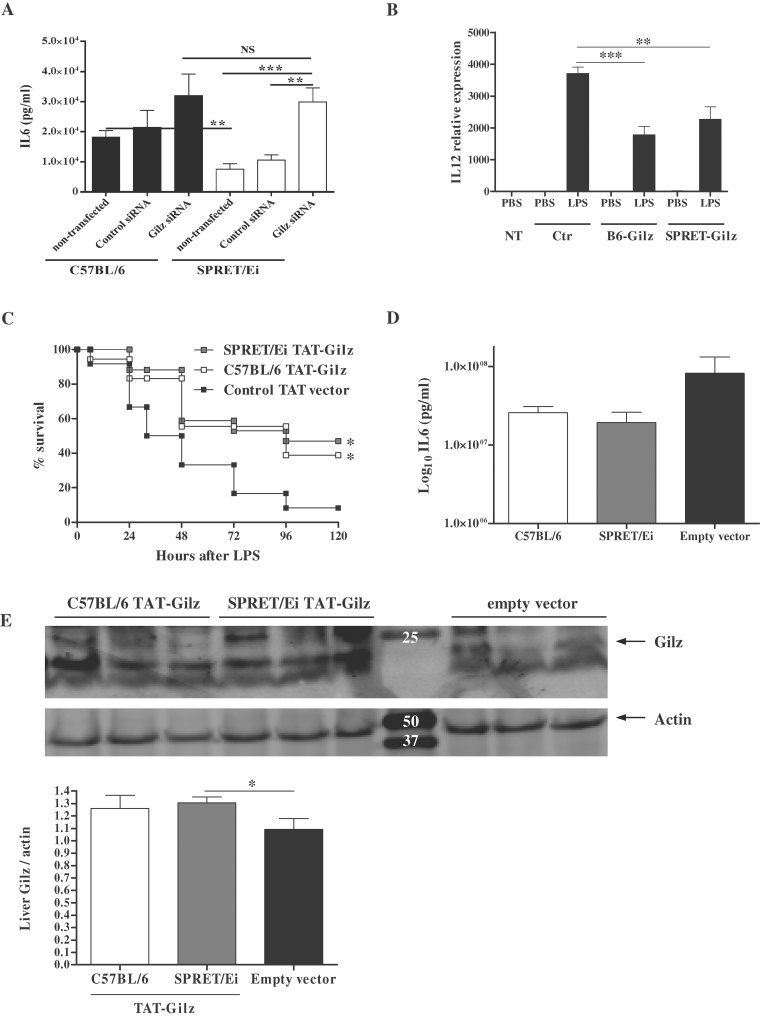
Increased levels of Gilz protect against the lethal effects of LPS **A,B.** Gilz expression is correlated with cytokine production in macrophages. Bone-marrow derived macrophages from C57BL/6 (n = 4) and SPRET/Ei (n = 4) mice (2 mice per genotype were used to generate BMDM) were either non-transfected or transfected with control or Gilz siRNA. After 7 days of culture, cells were stimulated with 100 ng/ml LPS for 6 h and IL6 measured in the supernatant **(A)**. Different pES34 plasmids (1 µg DNA), either control TAT or Gilz-containing (C57BL/6 or SPRET/Ei sequence) TAT-plasmids, were overexpressed in RAW264.7 macrophages (n = 5-6) and treated with PBS or 100 ng/ml LPS. 24 h after stimulation cells were harvested for RNA isolation and QPCR analysis of IL12 **(B)**. Results from graphs **(A)** and **(B)** were confirmed in two independent experiments and pooled data are shown here. In graphs **(A)** and **(B)**, data are expressed as mean ± s.e. and significances were calculated using Mann Whitney test. *, ** and *** represent *P* < 0.05, *P* < 0.01 and *P* < 0.001, respectively.**C,D.** Hydrodynamic injection of the pES34-TAT-Gilz vectors protects mice against endotoxemia. Female C57BL/6 mice received a hydrodynamic injection in the tail vein, either with a TAT-Gilz plasmid containing the coding sequence either from C57BL/6 (n = 18) or from SPRET/Ei (n = 17), or with the empty TAT plasmid (n = 12) as negative control. Mice were injected with 1 µg plasmid per gram of body weight followed 2 h later by i.p. injection of 350 µg of LPS. Mortality was monitored for five days, after which no further deaths occurred. This graph represents data combined from three independent experiments. *, p < 0.05, significances were calculated with a logrank (Mantel-Cox) test **(C)**. Six hours after LPS injection, blood was taken and serum IL6 levels were determined **(D)**.**E.** Hydrodynamic injection of the pES34-TAT-Gilz plasmid results in higher Gilz protein levels in the liver. Female C57BL/6 mice received a hydrodynamic injection in the tail vein, either with a pES34-TAT-Gilz plasmid containing the C57BL/6 (n = 5) or SPRET/Ei (n = 5) coding sequence, or with empty TAT plasmid (n = 5) as negative control. Four hours later, livers were isolated for protein determination of Gilz and of β-actin for normalization. Western blots for Gilz (15 kDa) and β-actin (42 kDa) are shown above, and the normalized Gilz protein levels are depicted below. Normalization was done by calculating the intensity ratio between Gilz and β-actin, and by normalizing for gel differences by taking the average intensity of repeated samples. These high-pressure experiments were performed three times and representative results from one experiment are shown.

Gilz has been reported to reduce the *in vitro* induction of inflammatory cytokines caused by LPS stimulation of cells (Eddleston et al, 2007). As we found that the significantly reduced cytokine production after LPS stimulation in SPRET/Ei is also found at the level of macrophages (*p* = 0.0096) (Fig 1E), we tested the causal role of Gilz in the LPS resistant phenotype of SPRET/Ei mice by performing knock down studies, using control and Gilz siRNA (Eddleston et al, 2007), in bone marrow derived macrophages derived from C57BL/6 and SPRET/Ei mice. We found that BMDM from SPRET/Ei compared to BMDM from C57BL/6 mice, produced significantly less (*p* = 0.0096) IL-6 6 hours after LPS stimulation. Specific Gilz knock down resulted in an increase in IL6 induction by LPS, both in C57BL/6 and SPRET/Ei macrophages, which annihilated the difference in LPS-induced IL-6 between these two species (*p* = 0.8113) (Fig 4A). This result suggests that Gilz indeed plays an essential role in the suppression of LPS-induced inflammation in SPRET/Ei macrophages.

An important role for Gilz as an anti-inflammatory molecule was further proven by transfection of different Gilz pES34 plasmids, expressing the Gilz sequence of either C57BL/6 or SPRET/Ei (107E > Q), in the mouse macrophage cell line RAW264.7. Compared to the control plasmid, expression of both Gilz versions resulted in significantly reduced induction of pro-inflammatory cytokines, such as IL12, after 24 hours of LPS stimulation (Fig 4B; *p* < 0.0001 and *p* = 0.0044 for C57BL/6 and SPRET/Ei Gilz plasmids, respectively). As this reduction was similar after transfection with both plasmids, this result suggests that the amino acid variation present in the SPRET/Ei sequence of Gilz does not lead to a change in specific activity of Gilz.

Furthermore, we examined whether Gilz administration by injecting female C57BL/6 mice with Gilz-expressing plasmids using high-pressure hydrodynamic plasmid injection, can protect against LPS *in vivo*. We used the same fusion constructs TAT-Gilz^B^ or the TAT-Gilz^S^ as in the *in vitro* experiments, containing the TAT peptide followed by the Gilz cDNA sequence from C57BL/6 and SPRET/Ei, respectively. In contrast with the empty TAT-containing vector, both TAT-Gilz vectors reduced mortality (Fig 4C) and IL6 serum concentrations to the same extent (Fig 4D), indicating that the SPRET/Ei Gilz allele does not confer extra protection against LPS. In addition, levels of Gilz were higher in livers of mice treated with one of both TAT-Gilz vectors compared to mice injected with the empty TAT vector (Fig 4E). This indicates that higher levels of Gilz are correlated with increased protection against the lethal effects of LPS. Again, there was no difference in Gilz protein levels between mice treated with TAT-Gilz^B^ or with TAT-Gilz^S^ (Fig 4E), suggesting that the coding region of SPRET/Ei Gilz allele does not contribute to increased Gilz levels in SPRET/Ei mice. These data support that the effect from Gilz on the LPS resistance is regulatory in origin.

## DISCUSSION

Sepsis remains one of the leading causes of death in intensive care units (Vincent et al, 2006) and new therapeutic approaches are urgently needed (Riedemann et al, 2003). We previously showed that SPRET/Ei mice are highly resistant to the lethal effects of LPS (Mahieu et al, 2006), as well as to certain bacterial infections (Dejager et al, 2010a). LPS-induced lethal inflammation and bacterial infections are often used to study the pathogenesis of sepsis (Dejager et al, 2011). We believe that SPRET/Ei mice could be useful for gaining deeper understanding of the complex pathology of sepsis and for identifying novel therapeutic possibilities.

The great genetic diversity between *Mus spretus* and *Mus musculus* has already been instrumental in the identification of genetic loci that contribute to resistance phenotypes of *Mus spretus*-derived strains (Dejager et al, 2009), such as resistance to infection with *Salmonella typimurium* (Dejager et al, 2010a) and *Yersinia pestis* (Blanchet et al, 2011). Here, we identified two QTL determining the resistance of SPRET/Ei mice to LPS, one of which is located on the X chromosome. We confirmed the importance of the SPRET/Ei X chromosome for LPS resistance by two means. First, (BxS)F1 females, carrying one X chromosome from C57BL/6 and one from SPRET/Ei, are more resistant to LPS than their (BxS)F1 male counterparts. Second, (SxB)F1 males, carrying a SPRET/Ei X chromosome, were more resistant to LPS than (BxS)F1 males, carrying a X from the C57BL/6 parent. This indicates that the X chromosome confers part of the LPS resistance and not the Y chromosome, because the Y chromosome contains only a few genes involved mainly in spermatogenesis and not in immune functions (Libert et al, 2010). It also is commonly believed that female sex steroids enhance protective immune responses, whereas androgens have been shown to render male mice more susceptible to inflammatory insults (Knoferl et al, 2002; Wichmann et al, 1996; Zellweger et al, 1997). However, our results are not confounded with the effects of sex steroids because neither ovariectomy nor castration affected the LPS response in the (BxS)F1 mice.

We recently reported that the SPRET/Ei LPS resistance depends at least partly on GR actions. GR levels and transcriptional activity are higher in SPRET/Ei mice than in C57BL/6 mice (Dejager et al, 2010b). This is in agreement with the enhanced resistance of mice with increased GR gene dosage to endotoxic shock (Reichardt et al, 2000). The high GR mRNA levels in SPRET/Ei mice were proven to be the result of a sequence variation very close to the location of the GR-encoding gene *Nr3c1* on chromosome 18 (Dejager et al, 2010b). In our study, LPS resistance did not map on chromosome 18, so high GR levels are probably not related to LPS hyporesponsiveness. We believe that GR actions, rather than GR levels, contribute to the LPS resistance of SPRET/Ei mice and that a locus downstream of the GC/GR axis in the distal region of the X chromosome is involved. Moreover, it has been suggested that GR-mediated protection against LPS in SPRET/Ei mice is predominantly dependent on the transactivation properties of the GR, rather than on its transrepression actions (Dejager et al, 2010b). It is conceivable that a GC-inducible gene contributes to the ability of SPRET/Ei mice to suppress inflammation during endotoxemia, as it has become clear that GR-transactivated genes play a prominent role in the anti-inflammatory actions of the GR (Newton & Holden, 2007). Thus, we hypothesized that a GRE-gene located on the X chromosome mediates the GR anti-inflammatory action. Using *in silico* analysis, we searched for GRE-genes in the candidate region on the X chromosome and found 41 GRE-containing genes. Based on ChIP-Seq analysis, we found that only one of those 41 genes, *Tsc22d3*, contains a functional GRE. Note that this analysis was performed in a mouse hepatoma cell line and that GR-mediated actions are strongly cell-type specific (Gross & Cidlowski, 2008). Therefore, we cannot exclude the presence of other functional GR-inducible genes in the critical region on the X chromosome.

We considered *Tsc22d3* the obvious candidate for the LPS resistance of SPRET/Ei mice. First, it co-locates with the QTL identified on the X chromosome. Second, Gilz is strongly induced by GR (D'Adamio et al, 1997; Eddleston et al, 2007; Godot et al, 2006) and it has anti-inflammatory properties (reviewed in (Beaulieu & Morand, 2011)). As Gilz represses NF-κB (Di Marco et al, 2007), it contributes to the strong anti-inflammatory actions of the GR (Berrebi et al, 2003; Mittelstadt & Ashwell, 2001). For example, Gilz expression in airway epithelial cells and human alveolar macrophages is protective against LPS-induced inflammation (Eddleston et al, 2007; Gomez et al, 2010; Hoppstadter et al, 2012). However, most studies on Gilz were done *in vitro*, but the *in vivo* anti–inflammatory potential of Gilz has received little attention (Cannarile et al, 2009). Our results indicate that Gilz might also be protective in models of acute inflammation, such as endotoxemia.

Gilz is widely expressed in cells of the hematopoietic lineage (Ayroldi & Riccardi, 2009) and in several murine and human tissues (Cannarile et al, 2001) at relatively high levels under basal conditions (Berrebi et al, 2003). We previously reported that SPRET/Ei mice exhibit significantly higher levels of Gilz mRNA than C57BL/6 mice. This difference in expression was partly attributed to the high GR activity in SPRET/Ei mice, which was demonstrated by performing adrenalectomy (Dejager et al, 2010b). This study indicated that both protein and mRNA levels (of the two smallest isoforms (Bruscoli et al, 2010; Soundararajan et al, 2007)) of Gilz are significantly higher in (BxS)F1 females than in (BxS)F1 males. Gilz protein levels followed the same pattern of expression as their transcript, as reported by Cannarile *et al.* (Cannarile et al, 2001). Several genes located in the same cluster as Gilz showed equal expression in female and male (BxS)F1 mice, which means that an effect of X inactivation escape can be dismissed. Although several genes on the human X chromosome escape inactivation (Carrel & Willard, 2005), this is not common in mouse (Disteche, 1999). In humans, this event generally occurs in clusters, mainly next to the PAR regions, and next to the centromere. Many genes that escape X inactivation in humans are subject to X inactivation in the mouse, suggesting that X inactivation is more complete in the mouse (Disteche et al, 2002). Additionally, Gilz levels are similar in C57BL/6 females and males, indicating that Gilz is expressed from one allele in female mice. We believe that it is unlikely that Gilz escapes X inactivation and so we hypothesize that such escape is not responsible for the higher levels in female (BxS)F1 mice.

As expected, (BxS)F1 females and males also showed no difference in GR levels, as both sexes carry one SPRET/Ei and one C57BL/6 GR allele (Dejager et al, 2010b). Although female (BxS)F1 mice had slightly higher levels of corticosterone than (BxS)F1 males, this did not confer the higher expression levels of Gilz, because the expression of other typical GRE genes, such as *Dusp1* and *G6P*, did not differ between the females and males. Therefore, we exclude a role for GR levels and activity in the difference in Gilz levels between (BxS)F1 females and males. However, variation in Gilz transcription levels mapped to the *Tsc22d3* locus on the X chromosome, suggesting that a *cis*-acting sequence variation leads to increased transcription of Gilz. Hence, we searched for variations in the SPRET/Ei *Tsc22d3* locus based on the available Sanger sequences. Many sequence variations (about 1 SNP every 100 bp) are expected because of the high genetic divergence between *Mus spretus* and *Mus musculus* (Dejager et al, 2009; Guenet & Bonhomme, 2003). In total, we identified six SNPs in the coding sequence, one of which leads to the substitution of the E at position 107 of the canonical isoform by a Q in SPRET/Ei mice. The high expression levels of Gilz mRNA can also be caused by sequence variations in the 3'UTR, the promoter region, or any other region affecting transcription and translation efficacy, promoter activity or exon splicing. Note that we do not mention the sequence variants for the other isoform and that these might also contribute to the differences in Gilz levels. In addition, by comparing the sequence variants in the SPRET/Ei *Tsc22d3* genomic sequence with sequences in LPS sensitive mouse strains, we found that the variants are unique for the SPRET/Ei strain. Further functional research is required to identify the causative polymorphism(s) that contribute to the higher expression levels of Gilz in SPRET/Ei.

Gilz is an important mediator of GR-driven anti-inflammatory actions (Ayroldi & Riccardi, 2009). Evidence shows that Gilz inhibition dampens the efficacy of GCs in different inflammatory models. For example, Gilz siRNA abrogates the effects of GC treatment on LPS-induced pro-inflammatory cytokines in circulating monocytes collected from alcoholic hepatitis patients (Hamdi et al, 2007), and Gilz siRNA increases disease severity in a mouse model of arthritis (Beaulieu et al, 2010). Although Suarez *et al.* recently reported that Gilz deficiency does not affect cytokine production after LPS stimulation of macrophages (Suarez et al, 2012), several other studies have reported the importance of Gilz as a powerful suppressor of inflammation in mice and in cells, such as macrophages (Hoppstadter et al, 2012) and airway epithelial cells (Eddleston et al, 2007) (reviewed in (Beaulieu & Morand, 2011), (Ayroldi & Riccardi, 2009)). An explanation for the discordant findings is currently difficult to give. We have shown in our study by overexpression and knock down studies in macrophages, that Gilz clearly significantly controls the production of cytokines after LPS.

Our results indicate that a *cis*-acting sequence variation in the *Tsc22d3* gene leads to increased Gilz levels and contributes to the resistance of SPRET/Ei mice to LPS. To determine whether increased Gilz levels might have a protective role in endotoxemia, we used pES34-TAT-Gilz expression vectors. The TAT-Gilz fusion vector and protein have already been used *in vitro* and *in vivo*. For example, it has been reported that TAT-Gilz associates with p65 and p52, showing that Gilz interacts directly with NFκB (Ayroldi et al, 2001). *In vivo*, TAT-Gilz protects mice against the development of DNBS-induced colitis (Cannarile et al, 2009). Our data indicate that macrophages transfected with TAT-Gilz, containing either the C57BL/6 or SPRET/Ei sequence, showed reduced cytokine production. In addition, mice treated with pES34-TAT-*Gilz* plasmids become resistant to the lethal effects of LPS due to the increased levels of Gilz. However, the resistance of these mice is not as strong as that of SPRET/Ei mice (Mahieu et al, 2006), which confirms the involvement of other genetic loci. Note that hydrodynamic tail vein injection results in specific uptake of plasmids in hepatocytes (Herrero et al, 2011; Wolff & Budker, 2005). Although LPS mainly activates macrophages and dendritic cells (Beutler & Rietschel, 2003), a role for the liver in the response to endotoxemia is certainly present (Heyninck et al, 2003). It was also reported by our group that GC-induced protection against LPS-induced shock is strongly mediated by hepatic GR (Van Bogaert et al, 2011). Since Gilz is induced by GR, the liver is likely involved in the protective capacities of Gilz. Moreover, administration of both pES34-TAT-*Gilz* vectors encoding either the C57BL/6 or the SPRET/Ei Gilz resulted in equal Gilz protein levels and in equal protection against LPS, suggesting that the sequence variant (107 E > Q) does not lead to higher Gilz levels nor to increased specific activity of Gilz, at least under these conditions.

In conclusion, we demonstrate a new anti-inflammatory role for Gilz in LPS-induced inflammation. This protective role of Gilz should be tested in more relevant models of sepsis, such as CLP (Dejager et al, 2011), but we believe that anti-inflammatory properties of Gilz could form the basis of novel therapeutic approaches for sepsis. Our results collectively underscore the contribution of GR transactivation to the anti-inflammatory effects of GR. This should be taken into account when designing dissociating GR ligands, *i.e.* reducing the transactivation actions of GR while maintaining its transrepression properties (Newton & Holden, 2007).

## MATERIALS AND METHODS

### Mice

C57BL/6J mice were purchased from Janvier (Le Genest-Saint-Isle, France). SPRET/Ei mice were obtained from The Jackson Laboratories (Bar Harbor, ME) and bred in our facility. We crossed C57BL/6 (B) females and SPRET/Ei (S) males to obtain (BxS)F1 progeny. Genetically, female and male (BxS)F1 mice differ only in their sex chromosomes. The reciprocal cross, *i.e.* SPRET/Ei females crossed with C57BL/6 males, was very unproductive, likely due to imprinting issues (L'Hote et al, 2010; Zechner et al, 1996). To map the loci responsible for SPRET/Ei LPS resistance, an interspecific backcross between female (BxS)F1 mice and C57BL/6 males was set up. The mice were kept in individually ventilated cages under a constant dark-light cycle in a conventional animal house and received food and water *ad libitum*. All mice were used at the age of 8–12 weeks. Animal experiments were approved by the institutional ethics committee for animal welfare of the Faculty of Sciences, Ghent University, Belgium.

### Construction and use of the pES34-TAT-Gilz vector

TAT-Gilz expression vectors were kindly provided by Dr. Carlo Riccardi (University of Perugia, Italy). The Gilz cDNA sequence (*Tsc22d3* gene) was inserted into the TAT (transactivator of transcription) vector to produce an in-frame fusion protein. The TAT peptide (GRKKRRQRRRPQ) is a cell-penetrating peptide derived from the transactivator of transcription of human immunodeficiency virus. This peptide can be used to overcome the lipophilic barrier of the cell membrane and deliver large molecules and small particles inside cells. The TAT-Gilz construct was cloned into the prokaryotic expression vector pGEX-4T-2, under the control of a chicken actin promoter, containing a GST (glutathione S-transferase)-tag to produce an in-frame fusion protein. The purified pGEX-4T-2-GST-TAT-Gilz vector was used to introduce the SPRET/Ei variation that leads to a change from a glutamic acid (E) to a glutamine (Q) at position 107 in the GILZ C-terminal region. This was done by site directed mutagenesis by using the QuickChange Site-Directed Mutagenesis kit (Stratagene). The mutated plasmid was purified by using the Plasmid-Mini purification kit (Qiagen) and sequenced for confirmation. Next, the TAT-Gilz fusion construct, devoid of the GST-tag, was subcloned into the pES34 eukaryotic expression vector by PCR and enzyme restriction. Both versions of the pES34-TAT-Gilz were then purified and injected into mice as described below.

### Endotoxemia model

LPS from *Salmonella abortus equi* was purchased from Sigma–Aldrich (Saint Louis, USA). Mice were injected intraperitoneally with LPS in 0.25 ml of pyrogen-free phosphate-buffered saline (PBS) and mortality and rectal body temperature with an electronic thermometer (Comark Electronics, Littlehampton, UK) was followed. Blood for cytokine measurement was collected with a glass capillary tube from the retro-orbital plexus 6 h after LPS injection. Blood was allowed to clot overnight at 4°C and serum was prepared by centrifugation at 20,000 g for 4 min and frozen at −20°C.

### *In vitro* stimulation and transfection of bone marrow-derived macrophages (BMDM) or RAW264.7 cells

Bone marrow cells were isolated from femurs and tibias of mice. To derive macrophages, after centrifugation (5 min, 1200 rpm, 4°C) cells were resuspended in 50 ml DMEM (Invitrogen) supplemented with 10% fetal calf serum, 100 units/ml penicillin/streptomycin, 2 mM L-glutamine, 1 mM sodium pyruvate, and 20 ng/ml mouse recombinant macrophage colony-stimulating factor. Bone marrow cells were cultured in bacterial Petri dishes for 7 days, and every 2 days the medium of the adherent cells was refreshed. After 7 days of differentiation, bone marrow-derived macrophages were detached with endotoxin- and enzyme-free cell dissociation buffer (Sigma) and counted by trypan blue exclusion, and the final concentration was adjusted to 10^6^ cells/ml in DMEM medium supplemented with growth factors. Next, macrophages (BMDM or RAW264.7) were plated out in 6-well plates at 10^6^ cells/well, after which they were transfected with equal volume of different siRNA (Eddleston et al, 2007) or Gilz-containing plasmids, respectively, using lipofectamine (Sigma). Next, cells were stimulated with LPS (100 ng/ml) and at different time points after LPS, IL6 levels were measured in supernatant using a bioassay (see below).

### Hydrodynamic tail vein injection of plasmids

To study whether increased levels of Gilz protect against LPS, mice were injected in the tail vein over 5 sec with a plasmid solution (10 µg/ml in sterile, endotoxin-free PBS) in a volume equivalent to 10% of the body weight. This high-pressure hydrodynamic plasmid injection technique leads to transient expression of plasmid genes in livers of injected mice. Two hours later, mice were treated with LPS and deaths were monitored. Mice were injected with TAT-Gilz expression vectors containing the Gilz coding sequence from C57BL/6 or SPRET/Ei, or a control TAT-containing vector.

**The Paper Explained**

PROBLEM:

Sepsis remains an important cause of death in intensive care units despite the availability of various therapeutics, such as glucocorticoids (GCs). The occurrence of about 750,000 cases of severe sepsis annually in the United States alone underscores the need for further extensive research. A commonly used model for studying the pathogenesis of sepsis is the LPS-induced endotoxemia model.

RESULTS:

SPRET/Ei mice, derived from the *Mus spretus* species, are remarkably more resistant to LPS injection than the commonly used laboratory C57BL/6 mice. Linkage analysis showed that the LPS resistance of SPRET/Ei mice maps to the distal part of chromosome X. Based on previous findings indicating that glucocorticoid receptor (GR) activity contributes to this resistance, we confirmed that Gilz is a GR-dependent gene co-locating with the QTL on the X chromosome. Next, we showed that increased levels of Gilz confer resistance to LPS, indicating that it plays an important role in the protection against endotoxemia.

IMPACT:

We provide the first evidence that Gilz has a strong anti-inflammatory role in acute inflammation. More specifically, increased levels of Gilz enhance the protection against the harmful effects of LPS. Gilz might turn out to be a good option for the treatment of acute inflammatory conditions such as sepsis.

### Cytokine measurements

Serum IL6 was determined with a 7TD1 hybridoma cell line as described (Vansnick et al, 1986). Serum levels of several cytokines were measured using Luminex technology (Bio-Rad, Nazareth-Eke, Belgium), following the manufacturer's protocol. Data were acquired using the Bio-Plex suspension array system (Luminex), a dual-laser, flow-based, microplate reader system.

### Ovariectomy and orchiectomy

Female (BxS)F1 mice were anesthetized with ketamine (75 mg/kg) and xylazine (10 mg/kg). A small midline dorsal incision was made in the skin, followed by flank incisions in the peritoneum. The ovaries were exteriorized, and the vessels attached to the ovaries were ligated and cut. Ovaries were then removed, and the peritoneum and skin were sutured. The same procedure was used on five other (BxS)F1 females, but ovaries were just exteriorized and then placed back (sham controls). For orchiectomy, male (BxS)F1 mice were anesthetized and a small abdominal incision was made in the skin and peritoneum. The testes and connected fat pad were exteriorized. After ligation, the arteries were cut, and the testes and epididymis were removed. Once the procedure was completed on both sides, the peritoneum and the skin were sutured. All animals were given antibiotics for one week. Success of ovariectomy and orchiectomy was determined by measuring estradiol and testosterone levels, respectively, by appropriate radioimmunoassays (Diasorin; Siemens Med.)

### Quantitative trait loci (QTL) mapping

Death or survival of the 140 BSB backcross offspring (61 females and 79 males) was monitored for five days after injecting with 250 µg of LPS. All backcross mice were genotyped using 87 microsatellite markers, the genome coverage of which was assessed by their position on the Mouse Genome Informatics database genetic map from The Jackson Laboratory and by applying a swept radius of 20 cM.

In addition, another backcross was set up and 172 offspring were obtained from the interspecific cross between female (BxS)F1 mice and male C57BL/6 mice. RNA was extracted from the livers of these mice at the age of 8 weeks. cDNA was synthesized and used for quantitative real time PCR analysis for Gilz (see qPCR below). The loci responsible for the increased levels of Gilz mRNA in SPRET/Ei mice were mapped using 81 microsatellite markers. In addition, 111 offspring were genotyped at two coding synonymous single nucleotide polymorphisms (T > C at 137075500bp and C > G at 137075502bp), together generating a *Pst*I restriction site (CTGGA/A > CTGCA/G) at the SPRET/Ei *Tsc22d3* allele. We made use of the Sanger sequences of the SPRET/Ei genome (http://www.sanger.ac.uk/) to identify the sequence variations between *Mus musculus* and *Mus spretus* at the Gilz locus.

We used linear mixed models in GenStat 14 (www.vsni.co.uk/software/genstat/) to explore the QTL for LPS resistance and Gilz expression levels. Gilz expression levels were log transformed before analysis. In a preliminary search for QTL, we tested the association of individual loci with the trait at every marker position along the genome, using the commonly known Simple Interval Mapping (SIM) procedure. Next, we searched for QTL at particular positions after correcting for QTL elsewhere in the genome, that were identified in the preliminary analysis. This procedure is commonly known as Composite Interval Mapping (CIM). The genome-wide type I error rate for detection of QTL co-segregating with the phenotypic trait was set to α = 0.05, corresponding to a threshold of (–log_10_(*P*) = 3.086).

### QPCR

Tissues samples were collected in RNA Later (Qiagen), and RNA was isolated with the RNeasyMini kit (Qiagen). RNA concentration was measured with the Nanodrop 1000 (Thermo Scientific), and cDNA was prepared by reverse transcription with the iScript cDNA synthesis kit (Bio-Rad). qPCR was performed using the Roche LightCycler 480 system (Applied Biosystems). Results are given as relative expression values normalized to the geometric means of the housekeeping genes.

### Western Blot analysis

The organs collected for western blotting were snap-frozen in liquid nitrogen and kept at –20°C. Later, they were homogenized in liquid nitrogen and cells were lysed in 300 µl of buffer containing 0.5% NP40 to disrupt the cytoplasmic membrane. Protein concentration was determined by using the DC Protein Assay kit (Bio-Rad). Whole tissue lysates (30 µg of lung lysate and 60 µg of liver lysate) were separated by electrophoresis in 15% gradient SDS-polyacrylamide gels and by using Tris-tricine-SDS electrophoresis buffer (Bio-Rad). Gels were transferred to pre-equilibrated Immun-Blot PVDF Membranes (Bio-Rad) in Towbin buffer containing 15% methanol. Blotting was done overnight at 10 V and 4°C. Membranes were incubated for 2 h at room temperature with primary rabbit anti-human-GILZ antibody, which was kindly provided by Dr. Jane Eddleston and Dr. Bruce Zuraw (University of California, San Diego), and anti-mouse β-actin antibody (MP Biomedicals, Europe) for internal control. After washing the blots with PBS containing 0.1% Tween-20, they were incubated for 1 h at room temperature with anti-rabbit antibody (IRDye 800, LI 926-32211) and anti-mouse antibody (IRDye 680, LI 926-32220, Westburgh, The Netherlands). Normalization was done using the Odyssey 2.1 software by calculating the intensity ratio between Gilz and β-actin. To normalize for the difference between gels, an average intensity of the duplicate samples was calculated per gel. A normalization factor was then obtained by dividing the higher average intensity by the lower one and used to normalize all values.

### ChIP-seq in BWTG3 cells and ChIP on liver samples

BWTG3 cells were cross-linked in 1% PFA for 10 min at room temperature. The reaction was stopped with 0.125 M glycine. Cells were washed in PBS, lysed in RIPA lysis buffer, sonicated and then immunoprecipitated with rabbit anti-GR antibody (Santa Cruz Biotechnology). Immunoprecipitated material was collected by centrifugation, washed, and resuspended in 200 µl elution buffer (0.5 M NaHCO_3_, 10% SDS) supplemented with proteinase K (100 µg/ml) and incubated for 30 min at room temperature. The material was de-cross-linked by raising the incubation temperature to 65°C for 16 h. The recovered DNA was purified by the PCR purification kit (Qiagen). DNA libraries were sent to FASTERIS SA (www.fasteris.com) for sequencing on an Illumina Genome Analyzer IIx. The data were processed using the Illumina CASAVA 1.7 package. Regions of transcription factor binding were identified with the MACS peak caller. For the analysis, we selected the strongest peaks for each experiment, *i.e.*, peaks with a false discovery rate (FDR) < 20% and a 20-fold enrichment level (reads under peak relative to control). The peak locations were extracted and extended by 100 bp on either side.

C57BL/6 and SPRET/Ei mice were injected with PBS or 200 µg Dex (Sigma) and livers were excised 2 hours later. Liver samples were homogenized in cold PBS and cross-linked in 2% paraformaldehyde for 10 minutes at room temperature. The reaction was stopped with 1M glycine. Liver lysates were sonicated and then immunoprecipitated with rabbit anti-GR antibody (5 µg per IP; sc8992, Santa Cruz Biotechnology). The material was de-cross-linked by raising the incubation time to 65°C for 16 hours. The recovered DNA was purified by the PCR purification kit (Qiagen). qPCR was performed using the Roche LightCycler 480 system (Applied Biosystems). The Gilz primer pair were 5'-TCTTCATGCCACCATCATGT-3' and 5'-CCTGGAACAAAGCTGGACTC-3'.

### Statistics

Differences in survival rates of backcross animals were tested using a χ^2^ test. Survival rates of (BxS)F1 progeny after injection with different LPS doses were compared by Fisher's exact test in GraphPad Prism 5.0 software (GraphPad Software, San Diego, CA, USA). Survival curves (Kaplan-Meier plots) were compared by a logrank (Mantel-Cox) test in GraphPad Prism. Statistical significance of differences in gene expression as measured by qPCR, body temperature, cytokine levels and western blot intensities was inferred from Student's t-test or by one-way or two-way ANOVA in GraphPad Prism. Data are expressed as mean ± s.e.. *, ** and *** represent *P* < 0.05, *P* < 0.01 and *P* < 0.001, respectively.

## Supplementary material

As a service to our authors and readers, this journal provides supporting information supplied by the authors. Such materials are peer reviewed and may be re-organized for online delivery, but are not copy-edited or typeset. Technical support issues arising from supporting information (other than missing files) should be addressed to the authors.
