# Supplementary material for: LPS resistance of SPRET/Ei mice is mediated by Gilz, encoded by the Tsc22d3 gene on the X chromosome
Source: EMBO Mol Med. 2013 Mar 5;5(3):456–70. doi: 10.1002/emmm.201201683 (PMC3598084; doi:10.1002/emmm.201201683)
Supplement: Supplementary file 1 [file emmm0005-0456-SD1.pdf]

EMBO Molecular Medicine

LPS resistance of SPRET/Ei mice is mediated by Gilz, encoded by the Tsc22d3 gene on the X chromosome

Pinheiro, Iris, Dejager, Lien, Petta, Ioanna, Vandevyver, Sofie, Puimège, Leen, Mahieu, Tina, Ballegeer, Marlies, Van Hauwermeiren, Filip, Riccardi, Carlo, Vuylsteke, Marnik, and Libert, Claude

Corresponding author: Claude Libert, Ghent University/VIB

Review timeline:

Submission date:	27 June 2012
Editorial Decision:	01 August 2012
Revision received:	16 November 2012
Editorial Decision:	06 December 2012
Revision received:	07 December 2012
Accepted:	12 December 2012

Editors: Anneke Funk / Natascha Bushati

Transaction Report:

(Note: With the exception of the correction of typographical or spelling errors that could be a source of ambiguity, letters and reports are not edited. The original formatting of letters and referee reports may not be reflected in this compilation.)

1st Editorial Decision 01 August 2012

Thank you for the submission of your manuscript "LPS resistance of SPRET/Ei mice is determined by Gilz, encoded by the Tsc22d3 gene on the X chromosome" to EMBO Molecular Medicine. We have now heard back from the three referees whom we asked to evaluate your manuscript. You will see that they find the topic of your manuscript potentially interesting. However, they also raise significant concerns on the study, which should be addressed in a major revision of the manuscript.

In particular, reviewer #2 highlights that the evidence supporting that Gilz is a major factor determining LPS resistance should be strengthened and makes specific suggestions to address this point. This reviewer also raises concerns about Gilz transgene delivery that should be convincingly addressed.

Given the balance of these evaluations, we feel that we can consider a revision of your manuscript if you can convincingly address the issues that have been raised within the space and time constraints outlined below.

Revised manuscripts should be submitted within three months of a request for revision. They will otherwise be treated as new submissions, unless arranged otherwise with the editor.

I look forward to seeing a revised form of your manuscript as soon as possible.

***** Reviewer's comments *****

Referee #1:

Libert and colleagues have identified 2 QTLs that account for the LPS resistance of SPRET/Ei mice. Here they show that one locus corresponds to the gene encoding Gilz, a known glucocorticoid induces anti-inflammatory protein that negatively regulates NFkB. These are important results suitable for publication in EMM as is.

Referee #2:

The LPS resistance of Spret/Ei mice, an important role for GR and increased expression of Gilz in the liver was shown before by the same group. As GR can induce Gilz expression, the data suggested that Gilz may be a mediator of GR-dependent resistance to LPS. In this manuscript, the authors identify a QTL for resistance to LPS on the x-chromosome and provide evidence that genetic variation in the Gilz locus underlies the increased resistance of Spret/Ei mice to LPS. In contrast to the earlier work, this study suggests that a GR-independent increase in Gilz expression makes a major contribution to the Spret/Ei phenotype. In addition to a large number of non-coding SNPs in the Tsc22d3 locus, the Spret/Ei version of Gilz contains a amino acid substitution which is however suggested to be functionally equivalent to the B6 version based on an overexpression experiment in mice. The study nicely shows the utility of the Spret/Ei mice for candidate gene identification in backcross analyses. However, I am not completely convinced that Gilz is the major player in the LPS resistance of Spret/Ei mice, mainly because there are no Gilz knockout or knockdown data. In addition, the differences in expression are very moderate.

Specifically, I have the following comments:

Major points:
1. Confirmation of the LPS resistance in macrophages in vitro should be tried using macrophages from BxS and SxB male mice. Important to define whether inhibition of IL-6 production occurs in a cell-autonomous manner.
2. If the phenotype of impaired cytokine production is found in macrophages, Gilz expression could be knocked down by siRNA to directly prove an essential role for Gilz in Spret/Ei macrophages.
3. The phenotype of the recently published Gilz knockout mice (Suarez 2012 Mol Endocrinology) argues against an important role for Gilz in the control of cytokine production by macrophages and DC in response to TLR ligands or Candida. In addition, the suppressive effect of Dexamethasone on these inflammatory cytokines was completely unaltered in Gilz knockout macrophages. How can these divergent results be reconciled with the authors' data?
4. Coding sequence variation does not explain the difference in Gilz activity. There is a large number of SNPs in the 5 kB promoter region, but relatively few differences in the 3' UTR. As the suggested mechanism of LPS resistance is increased Gilz mRNA expression in Spret/Ei mice it would be important to test in reporter gene assays whether the promoter and/or 3'UTR region confer increased basal or GR-induced expression.
5. There are several isoforms described for Gilz. Have the authors determined whether Spret/Ei mice differentially express particular isoforms?
6. Hydrodynamic plasmid injection leads to uptake and expression of the transgene predominantly in hepatocytes. However, LPS-induced cytokine production does not come from hepatocytes but from innate immune cells and endothelial cells. Therefore, Gilz has to be delivered to these cells. The authors used a system where Gilz was cloned into a TAT-fusion expression vector to generate a secreted version that is then expected to penetrate cells in the periphery. It would therefore be more convincing to show that Gilz is indeed detectable in other organs that significantly contribute to LPS-induced cytokine release, e.g. spleen, lung or bone marrow.
7. The effect of TAT-Gilz in vivo overexpression on LPS resistance is weaker than the phenotype of Spret/Ei mice. The authors discuss this difference and suggest that it indicates the action of other genetic loci. However, the overexpression of TAT-Gilz in the liver is not strong, and importantly it is unclear how much TAT-Gilz is delivered to LPS responsive cells throughout the body. It is therefore preliminary to conclude that the sequence variation 107E>Q does not affect the Gilz protein levels or activity. Overexpression of B6 and Spret versions of Gilz in macrophages in vitro should be done to corroborate the in vivo data.

8. Finally, for a potential role of Gilz in humans the question is important whether evidence exists for genetic variation in Gilz expression in humans.

Minor points:
1. The levels of IL-6 shown in Figures 1 and 4 are unbelievably high: 10e7 ng/ml equals 10 mg/ml serum, which would be a nice way to produce nearly crystalline cytokine. There must have occurred an error in the calculation.
2. The densitometry of Gilz Westernblot gels in Figure 2D does not match the gel pictures shown: in the liver, F1 females have much stronger Gilz bands than the males, in the lung no difference is apparent; however, densitometry results suggest a moderate but significant difference in both organs.
3. To me, it is questionable whether a 20% reduction in mRNA expression can be reliably measured by real-time PCR (Figure 2E).

Referee #3 (Comments on Novelty/Model System):

Experiments have been carefully performed on an animal model which is recognized and widely used to study pathophysiologic manifestations of sepsis.
The data presented in this paper show how changes in the level of expression of the Gilz gene can dramatically alter the susceptibility to LPS in this model, which could have interesting therapeutical impact.

Referee #3 (Other Remarks):

In this paper, Pinheiro et al. have identified a strong candidate to explain the resistance of SPRET/Ei mice to the injection of LPS, while other mouse strains, such as C57BL/6 succomb in a few hours from endotoxemia. Through a combination of genetic mapping, gene expression analysis, and functional testing, they show that Gilz, an important anti-inflammatory GRE gene, is expressed at higher levels in SPRET than in B6, and in females than in males, and that overexpression correlates with stronger resistance to LPS injection.
Altogether, the experimental data support convincingly this hypothesis, and, considering the already known importance of Gilz in inflammation, and although they have not yet identified the causative DNA variant, their work is clearly worth publishing since it shows how the level of expression of Gilz by itself can influence dramatically the susceptibility to LPS injection. This work could have promising therapeutical applications.
The major strength of this work are : the use of an experimental model relevant to sepsis and endotoxemia under well-controlled conditions; carefully designed and performed experiments; good interpretation of data obtained.

Several points deserve to the improved or clarified in the manuscript.
1- The title states leaves no uncertainty to the role of Gilz, while in the abstract one reads 'strongly suggest'. Although I am personally convinced by the role of Gilz, the formal proof would come from replacing B6 genomic sequence with SEG sequence. I recommend that the title be softened.
2- The strongest argument for the role of X-linked gene(s) comes from the QTL mapping of LPS susceptibility. All other experiments, which compare males and females, are consistent with a role for the X chromosome and are interesting but do not prove that X-linked genes are directly involved. The differences between males and females could be indirect consequences of sex-dimorphism. See for example page 5, the comparison between (BxS)F1 males and females.
3- Apparently, BSB mice of both sexes were analyzed simultaneoulsy. Was there any difference in survival between BSB males and females ? Was the QTL analysis performed also for each sex separately to ensure that the same QTLs are found in both sexes ? Is the X chromosome QTL effect similar in males and females, or stronger in one sex ?
4- Authors should not use B/B or B/S for the genotype of BSB males on the X chromosome (text and supporting figure 1). BSB males are either XB/YB or XS/YB.
5- B6 x spretus crosses are performed by crossing B6 females with spretus females, as the other direction rarely yields progeny. Can the authors comment and provide details in the materials and methods ? Also, F1s from reciprocal crosses do not differ only in the sex chromosomes (bottom

page 5). They also differ in imprinted genes (which may explain why reciprocal crosses are not always possible).
6- The experiment on ovx and castrated mice rules out the role of sex hormones in the higher resistance to LPS of females. But, again, it does not tell anything about X-linked genes. The last sentence of the second paragraph of page 6 should be amended.
7- Figure 3D does not show two close peaks on chr X as mentionned at the bottom of page 7. Grph shows a single peak. Also, it is unlikely to see two linked peaks at 2 cM interval in BC analysis.
8- Supporting figure 1 : the individual effect of each QTL is not provided. What is presented (39% and 55%) is the effect of one QTL in only one genotype group for the other QTL.
9- p7 : "This was true for C57BL/6 female mice compared to C57BL/6 males and (BxS)F1 FEMALES COMPARED TO males (Fig. 3B)."
10- page 16 "172 offspring obtained from an interspecific backcross between female (BxS)F1 mice and male C57BL/6 mice." should be moved to the beginning of the paragraph.
11- How was body temperature measured (method, device) ?

1st Revision - authors' response 16 November 2012

Referee #1:

Libert and colleagues have identified 2 QTLs that account for the LPS resistance of SPRET/Ei mice. Here they show that one locus corresponds to the gene encoding Gilz, a known glucocorticoid induces anti-inflammatory protein that negatively regulates NFkB. These are important results suitable for publication in EMM as is.

Thank you for this positive comment.

Referee #2:

The LPS resistance of Spret/Ei mice, an important role for GR and increased expression of Gilz in the liver was shown before by the same group. As GR can induce Gilz expression, the data suggested that Gilz may be a mediator of GR-dependent resistance to LPS. In this manuscript, the authors identify a QTL for resistance to LPS on the x-chromosome and provide evidence that genetic variation in the Gilz locus underlies the increased resistance of Spret/Ei mice to LPS. In contrast to the earlier work, this study suggests that a GR-independent increase in Gilz expression makes a major contribution to the Spret/Ei phenotype. In addition to a large number of non-coding SNPs in the Tsc22d3 locus, the Spret/Ei version of Gilz contains a amino acid substitution which is however suggested to be functionally equivalent to the B6 version based on an overexpression experiment in mice. The study nicely shows the utility of the Spret/Ei mice for candidate gene identification in backcross analyses. However, I am not completely convinced that Gilz is the major player in the LPS resistance of Spret/Ei mice, mainly because there are no Gilz knockout or knockdown data. In addition, the differences in expression are very moderate.

Specifically, I have the following comments:

Major points:
1. Confirmation of the LPS resistance in macrophages in vitro should be tried using macrophages from BxS and SxB male mice. Important to define whether inhibition of IL-6 production occurs in a cell-autonomous manner.

Unfortunately, the SxB cross is extremely unproductive. During more than five years of crossing, we only obtained 4 offspring mice from this cross. We also tried IVF and even iPS approaches, but in vain. The few mice that we obtained by natural crosses mice were used in the LPS lethality experiment that is shown in figure 1F and 1G. However, as a worthy alternative, we now measured IL6 production in macrophages (BMDM) derived from (BxS) females and males and from C57BL/6 mice and included this figure in the revised manuscript (Fig. 1E). From these data, we can conclude that the significantly reduced IL6 production that we observed in (BxS)F1 females is also observed

in macrophages, and hence occurs in a cell-autonomous way. These data are included and discussed in the new paper.

2. If the phenotype of impaired cytokine production is found in macrophages, Gilz expression could be knocked down by siRNA to directly prove an essential role for Gilz in Spret/Ei macrophages.

We agree that this is a very informative experiment and have performed experiments in that direction. We performed two independent experiments in which Gilz was knocked down using siRNA in bone marrow derived macrophages from C57BL/6 and SPRET/Ei mice. We have pooled the results of both experiments. It is clear that the LPS-induced IL6 levels are lower in SPRET/Ei cells compared to C57BL/6 cells. However, when Gilz levels were reduced by knock-down, IL6 levels increased in both the C57BL/6 cells as the SPRET/Ei cells and reached similar amounts in both genotypes. This illustrates that Gilz is responsible for the reduced cytokine production in SPRET/Ei cells. These data were included and discussed in the revised manuscript (Fig. 4A).

3. The phenotype of the recently published Gilz knockout mice (Suarez 2012 Mol Endocrinology) argues against an important role for Gilz in the control of cytokine production by macrophages and DC in response to TLR ligands or Candida. In addition, the suppressive effect of Dexamethasone on these inflammatory cytokines was completely unaltered in Gilz knockout macrophages. How can these divergent results be reconciled with the authors' data?

It is true that Suarez et al. recently reported that Gilz deficiency in BMDM does not affect cytokine production after TLR stimulation. These authors also report that dexamethasone-mediated suppression of cytokine secretion was unaffected by the loss of Gilz. Owing to the redundant immunosuppressive role of GR and Gilz (via transrepression of NFkB), it is conceivable that stimulation of GR results in the reduction of cytokine production. Therefore, we believe that, based on these data, you cannot completely exclude a role for Gilz in the innate immune regulation. Furthermore, this manuscript is in contrast to many other publications that show the importance of Gilz in both innate and adaptive immune functions, i.e. Gilz is well known to interfere with NFκB-mediated gene transcription in several immune cells. For example, Hoppstäder et al. (2012) recently showed that Gilz plays a prominent role in the anti-inflammatory actions of GCs in alveolar macrophages. Using Gilz knockdown approaches, they obtained a pro-inflammatory response, as indicated by increased cytokine production and NFkB activity. In addition, they show that Gilz levels are down regulated upon TLR activation, a mechanism which likely accounts for an increased immune response. In addition, Eddleston et al. (2007) reported a prominent role for Gilz in the anti-inflammatory actions of GR in airway epithelial cells. In conclusion, from carefully searching through literature, there is a multitude of studies showing anti-inflammatory effects of Gilz and several studies that convincingly show that Gilz mediates at least part of the anti-inflammatory effects of GCs (reviewed in Ayroldi et al. 2009; Beaulieu et al. 2011). The Suarez paper is not able to confirm these findings. It is clear that there is some controversy about the anti-inflammatory effects of Gilz, but based on our work, we believe that the increased basal levels of Gilz in SPRET/Ei mice result in the enhanced protection against LPS-induced lethality. We included these reflections in the discussion of the new manuscript.

4. Coding sequence variation does not explain the difference in Gilz activity. There is a large number of SNPs in the 5 kB promoter region, but relatively few differences in the 3' UTR. As the suggested mechanism of LPS resistance is increased Gilz mRNA expression in Spret/Ei mice it would be important to test in reporter gene assays whether the promoter and/or 3'UTR region confer increased basal or GR-induced expression.

It would indeed be interesting to identify the causative polymorphism(s) that contribute to the higher expression levels of Gilz in SPRET/Ei. However, due to time limitations we decided not to make different reporter constructs using the promoter region and 3'UTR of the SPRET/Ei Gilz sequence. The basic message of our paper is to show the linkage of the great LPS resistance of SPRET/Ei to Gilz. The details as to which sequence variation is really causative will be studied later, but we do not really consider this as top-priority or even feasible at all, given the great amount of sequence polymorphisms in the Gilz locus of SPRET/Ei.

5. There are several isoforms described for Gilz. Have the authors determined whether Spret/Ei mice differentially express particular isoforms?

Indeed, there exist several isoforms of Gilz, 4 protein-coding and 3 non-protein-coding ones (Soundararajan et al. 2007; Bruscoli et al. 2010). The primers that were used for the QPCR analysis in figure 2C are not specific for a single transcript variant, as they measure all the variants at once. As suggested by the reviewer, we analyzed the expression of Gilz transcript variants in the lungs and livers of (BxS)F1 females and (SxS)F1 males. We could only detect the two smallest isoforms of Gilz (113 and 134 amino acids) (Fig. 2C). It is known that the expression of different transcriptional variants varies among tissues. Interestingly, the higher expression of Gilz in females compared to males is true for both these isoforms. This result is now displayed as Fig. 2C and discussed in the results and discussion sections.

6. Hydrodynamic plasmid injection leads to uptake and expression of the transgene predominantly in hepatocytes. However, LPS-induced cytokine production does not come from hepatocytes but from innate immune cells and endothelial cells. Therefore, Gilz has to be delivered to these cells. The authors used a system where Gilz was cloned into a TAT-fusion expression vector to generate a secreted version that is then expected to penetrate cells in the periphery. It would therefore be more convincing to show that Gilz is indeed detectable in other organs that significantly contribute to LPS-induced cytokine release, e.g. spleen, lung or bone marrow.

Hydrodynamic tail vein injection indeed results in specific uptake of the plasmid in hepatocytes (Wolff et al. 2005; Herrero et al. 2011). Consistently, higher Gilz expression levels were only observed in the liver, and not in spleen or lung. Although it is true that LPS mainly activates macrophages and dendritic cells to stimulate the innate immune response (Beutler et al. 2003), a role for the liver cannot be underestimated (Jirillo et al. 2002). For example, NFkB activation in hepatocytes plays an important role in LPS-induced liver failure (Heyninck et al. 2003). Also, glucocorticoid-induced protection against LPS- or TNF-induced lethal shock is strongly mediated by hepatic GR, as we found using hepatocyte-specific GR knockout mice (Van Bogaert et al., JBC, 2011). Since we consider Gilz as a molecule, induced by GR, we felt that the liver might, at least partly be involved in the protective capacities of Gilz. In view of the true goals of this part of the study, namely (1) to demonstrate that increased Gilz leads to reduced LPS response and (2) to demonstrate that no obvious differences are found between the B and S form of Gilz, this fast, easy and informative way of plasmid delivery was used in the paper.
We have motivated this choice better in our paper in the discussion section, also in view of the Gilz overexpression experiments using mouse macrophages (Fig. 4B). Because we are also interested in the issue that the reviewer raises here, we have initiated a new project, namely the transgenic overexpression of Gilz in the ROSA26 locus of mice, in a conditional way. The targeting vector has recently been transfected in ES cells and chimeric mice are born. We hope we will have germline transmission. The idea is to backcross the mutant mice into C57BL6 and then cross them with macrophage-specific (LysM) cre mice. The resulting mice will certainly be very informative for further endotoxemia and sepsis studies, but, if all goes well, these results will not be available in the next 2-3 years, which is of course far beyond the time-line of our *Spretus* paper.

7. The effect of TAT-Gilz in vivo overexpression on LPS resistance is weaker than the phenotype of Spret/Ei mice. The authors discuss this difference and suggest that it indicates the action of other genetic loci. However, the overexpression of TAT-Gilz in the liver is not strong, and importantly it is unclear how much TAT-Gilz is delivered to LPS responsive cells throughout the body. It is therefore preliminary to conclude that the sequence variation 107E>Q does not affect the Gilz protein levels or activity. Overexpression of B6 and Spret versions of Gilz in macrophages in vitro should be done to corroborate the in vivo data.

We agree with the reviewer. His/her suggestion to overexpress both the B and S version of the Gilz gene in macrophages is a very good suggestion, and we have taken it at heart. The mouse macrophage cell line RAW 264.7 was transfected with control plasmid, B6-Gilz-expressing plasmid and SPRET-Gilz-expressing plasmid and then stimulated with LPS and pro-inflammatory gene expression (e.g. IL12) was measured. Interestingly, both Gilz versions reduced the LPS-induced cytokine expression significantly, while there was no significant difference between the magnitude of effect of the B6 and SPRET versions of Gilz. This result is shown in the paper as Fig. 4B and is in accordance to the conclusion that both Gilz versions are equally potent. These results are interpreted in the discussion section of the revised paper.

8. Finally, for a potential role of Gilz in humans the question is important whether evidence exists for genetic variation in Gilz expression in humans.

To our knowledge and according to OMIM, there is nothing reported on genetic variation at the level of Gilz in humans, nor about linkage of Gilz with diseases.

Minor points:
1. The levels of IL-6 shown in Figures 1 and 4 are unbelievably high: 10e7 ng/ml equals 10 mg/ml serum, which would be a nice way to produce nearly crystalline cytokine. There must have occurred an error in the calculation.

This was indeed a mistake, this should have been pg/ml and has been changed accordingly.

2. The densitometry of Gilz Westernblot gels in Figure 2D does not match the gel pictures shown: in the liver, F1 females have much stronger Gilz bands than the males, in the lung no difference is apparent; however, densitometry results suggest a moderate but significant difference in both organs.

The Gilz bands were quantified using the most appropriate program (Odyssey) and normalized against the actin bands. Here you can find a table with the respective intensity values, which clearly shows that there are small but significant different Gilz levels in F1 females and males. We do not intend to show these values in the paper.

LIVER			LUNG	
F1 female	F1 male		F1 female	F1 male
1,508175	0,919867		0,674887	0,618996
1,405692	1,101333		0,622024	0,635239
1,382438	0,914704		0,639162	0,628461
1,445117	1,215512		0,610163	0,595847
0,966249	1,051379		0,701766	0,62363
1,09237	0,895964		0,666752	0,596559
0,870493	0,865491		0,657827	0,636612
1,063839			0,758618	

3. To me, it is questionable whether a 20% reduction in mRNA expression can be reliably measured by real-time PCR (Figure 2E).

If there is one expression technology able to detect a 20% difference between two treatments, it is real-time PCR beyond doubt. Gilz Ct values ranged from 27.6 up to 31.6, reflecting expression levels significantly different from background. In addition, mean values and s.e.'s are obtained from 5-6 replicates per genotype. The s.e.'s are rather small indicating little uncertainty in the data. Therefore, it is very unlikely that the 20% reduction in expression observed happened by chance.

Referee #3 *(Comments on Novelty/Model System):*

Experiments have been carefully performed on an animal model which is recognized and widely used to study pathophysiologic manifestations of sepsis.
The data presented in this paper show how changes in the level of expression of the Gilz gene can dramatically alter the susceptibility to LPS in this model, which could have interesting therapeutical impact.

Referee #3 *(Other Remarks):*

In this paper, Pinheiro et al. have identified a strong candidate to explain the resistance of SPRET/Ei mice to the injection of LPS, while other mouse strains, such as C57BL/6 succomb in a few hours from endotoxemia. Through a combination of genetic mapping, gene expression analysis, and functional testing, they show that Gilz, an important anti-inflammatory GRE gene, is expressed at higher levels in SPRET than in B6, and in females than in males, and that overexpression correlates with stronger resistance to LPS injection.
Altogether, the experimental data support convincingly this hypothesis, and, considering the already known importance of Gilz in inflammation, and although they have not yet identified the causative DNA variant, their work is clearly worth publishing since it shows how the level of expression of Gilz by itself can influence dramatically the susceptibility to LPS injection. This work could have promising therapeutical applications.
The major strength of this work are : the use of an experimental model relevant to sepsis and endotoxemia under well-controlled conditions; carefully designed and performed experiments; good interpretation of data obtained.

Several points deserve to the improved or clarified in the manuscript.
1- The title states leaves no uncertainty to the role of Gilz, while in the abstract one reads 'strongly suggest'. Although I am personally convinced by the role of Gilz, the formal proof would come from replacing B6 genomic sequence with SEG sequence. I recommend that the title be softened.
We agree that the use of consomic mice, introducing the chromosomal segment from SPRET/Ei *Tsc22d3* into a C57BL/6 background, would be very useful for the genetic dissection of multigenic traits into a series of monogenic characters. It would ultimately proof the role of the sequence variation and consequently higher Gilz levels in the LPS resistance of SPRET/Ei mice. However, after several attempts, we did not succeed in generating such mice. In fact, one is crossing individuals of different species, which causes many problems over the following generations, especially problems of lethality and infertility. Generating consomic B.S mice has proven only partially possible, as discussed L'Hôte et al. 2010. Our experience with generating B.S consomic or congenic mice involving the X-chromosome are disappointing and have not resulted in mice useful for this Gilz story. We have made a comment about this in the discussion of the paper.
As requested, we adjusted the title slightly.

2- The strongest argument for the role of X-linked gene(s) comes from the QTL mapping of LPS susceptibility. All other experiments, which compare males and females, are consistent with a role for the X chromosome and are interesting but do not prove that X-linked genes are directly involved. The differences between males and females could be indirect consequences of sex-dimorphism. See for example page 5, the comparison between (BxS)F1 males and females.

We partially agree with the reviewer. However, we must stress that also in bone-marrow derived macrophages, in the cultures of which there are no sex hormones, LPS induces significantly less inflammation in (BxS)F1 female than male cells (Fig. 1E) and that these differences are annihilated after knocking-down Gilz (Fig. 4A in the revised manuscript). These data, to our opinion support the genetic linkage data between LPS resistance and Gilz. These data are displayed and discussed in this context in the new version of the paper.

3- Apparently, BSB mice of both sexes were analyzed simultaneoulsy. Was there any difference in survival between BSB males and females ? Was the QTL analysis performed also for each sex separately to ensure that the same QTLs are found in both sexes ? Is the X chromosome QTL effect similar in males and females, or stronger in one sex ?

Linkage analysis for each sex separately detected both QTL on chromosomes 2 and X, though at a lower significance. This can be explained by the lower number of BSB individuals analyzed in each analysis separately (see table below). We included the results of these QTL analyses as Supporting Information Fig. 1B.

**females**	**61**	32	resistant	52	% survived
		29	susceptible	48	% died
			total	100	%
**males**	**79**	58	resistant	73	% survived
		21	susceptible	27	% died
				100	%
**total**	**140**	90	resistant	64	% survived
		50	susceptible	36	% died
				100	%

4- Authors should not use B/B or B/S for the genotype of BSB males on the X chromosome (text and supporting figure 1). BSB males are either XB/YB or XS/YB.

The figure includes both sexes in the calculations, that is why we haven't discriminated between the two. Actually, BSB males are not heterozygous for the X chromosome, but hemizygous. However, for calculation purposes they are considered heterozygous (also in mapping software program they are considered heterozygous because it does not allow a third "entry status" for the X chromosome). Indeed, males always have the Y chromosome from C57BL/6, so they are implicitly SB or BB (being the red the Y chromosome). Females can also only be SB or BB (as one X is always from the father C57BL/BL6), so that is why is depicted in this way.

5- B6 x spretus crosses are performed by crossing B6 females with spretus females, as the other direction rarely yields progeny. Can the authors comment and provide details in the materials and methods? Also, F1s from reciprocal crosses do not differ only in the sex chromosomes (bottom page 5). They also differ in imprinted genes (which may explain why reciprocal crosses are not always possible).

It is true that the reciprocal cross is extremely unproductive. During more than five years of crossing, we only obtained 4 offspring mice from this cross. We also tried IVF and even iPS approaches to yield more mice, but in vain. Parent-of-origin imprinting could indeed be a possible reason for the invariable sterility of reciprocal crosses. It has been shown that such crosses suffer from hyper- or hypoplastic placentas, compared with intraspecific crosses (Zechner et al. 1996 Nat Genet). Various degrees of placental hyperplasia or hypoplasia are caused by different interactions between autosomes and the X chromosome, depending on parent- and species-of-origin of the chromosomes (Zechner et al. 1997 Genetics). These issues are now mentioned in the materials and methods section of the corrected manuscript, as suggested by the reviewer.
In addition, we also adapted the paragraph concerning the mentioned genetic difference between male F1 mice from the reciprocal crosses.

6- The experiment on ovx and castrated mice rules out the role of sex hormones in the higher resistance to LPS of females. But, again, it does not tell anything about X-linked genes. The last sentence of the second paragraph of page 6 should be amended.

We agree with the reviewer and have corrected the text accordingly.

7- Figure 3D does not show two close peaks on chr X as mentioned at the bottom of page 7. Graph shows a single peak. Also, it is unlikely to see two linked peaks at 2 cM interval in BC analysis.

We agree that this is not well formulated in the initial version of the manuscript. Actually, in first instance, we genotyped all the mice for several markers divided across the genome, including marker DXMit34, with which we observed linkage. As this marker is located 2 cM from the *Tsc22d3* locus, we genotyped half of the backcross mice (only half due to a lack of DNA left for the others) also for a marker located in the *Tsc22d3* region. So, in fact, there is linkage to the indicated marker on chromosome X in Figure 3D and likely to the *Tsc22d3* locus. This was corrected in the text.

8- Supporting figure 1 : the individual effect of each QTL is not provided. What is presented (39% and 55%) is the effect of one QTL in only one genotype group for the other QTL.

Indeed, the percentages in supporting figure 1 are misleading and, therefore, discarded. We added a table giving more details about the analysis performed to detect epistasis or the lack thereof. The survival data was modeled as binomial data, followed by fitting a generalized linear model, incorporating a logit link function, as implemented in Genstat. The conclusion from the accumulated summary is there are significant QTL effects, but no interaction, indicative for the lack of epistasis.

9- p7 : "This was true for C57BL/6 female mice compared to C57BL/6 males and (BxS)F1 FEMALES COMPARED TO males (Fig. 3B)."

This was corrected in the revised manuscript.

10- page 16 "172 offspring obtained from an interspecific backcross between female (BxS)F1 mice and male C57BL/6 mice." should be moved to the beginning of the paragraph.

We agree that this was not clearly written and we corrected the paragraph as suggested.

11- How was body temperature measured (method, device) ?

Rectal body temperatures were measured with an electronic thermometer (Comark Electronics, Littlehampton, UK). This information was added to the material and methods section of the revised manuscript.

2nd Editorial Decision 06 December 2012

Thank you for the submission of your revised manuscript to EMBO Molecular Medicine. We have now received the enclosed report from the referee whom we asked to re-assess it. As you will see the reviewer is now globally supportive and I am pleased to inform you that we will be able to accept your manuscript after the following editorial points have been addressed:

- The description of all reported data that includes statistical testing must state the name of the statistical test used to generate error bars and P values, the number (n) of independent experiments underlying each data point (not replicate measures of one sample), and the actual P value for each test (not merely 'significant' or 'P < 0.05').

Please see http://onlinelibrary.wiley.com/journal/10.1002/(ISSN)1757-4684/homepage/ForAuthors.html#data2 for more information.

- Please include a Table of Contents as the first page of your Supporting Information.

- We have noticed that in Figure 2D, the image of the Western blot is cropped to close to the bands. Please replace this with an image according to our guidelines: Cropped gels in the paper must retain all important bands, and space (several bandwidths) should be retained above and below the relevant band(s). Vertically sliced images that juxtapose lanes that were non-adjacent in the gel must have a clear separation or a black line delineating the boundary between the gels.

- We now encourage the publication of source data, particularly for electrophoretic gels and blots, with the aim of making primary data more accessible and transparent to the reader. Would you be willing to provide a PDF file per figure that contains the original, uncropped and unprocessed scans of all or key gels used in the figure? The PDF files should be labeled with the appropriate figure/panel number, and should have molecular weight markers; further annotation may be useful but is not essential. The PDF files will be published online with the article as supplementary "Source Data" files.

Please submit your revised manuscript within two weeks.

I look forward to reading a new revised version of your manuscript.

***** Reviewer's comments *****

Referee #2:

The authors have addressed my major concerns and performed experiments in macrophages including knockdown of Gilz in macrophages. The lack of phenotype in Gilz-deficient mice reported by others is still puzzling, but the reasons for the discrepant results will be sorted out in the future by the community.

2nd Revision - authors' response 07 December 2012

It is our pleasure to submit a revised version of our paper entitled "LPS resistance of SPRET/Ei mice is determined by Gilz, encoded by the Tsc22d gene on the X chromosome" (EMM-2012-01683-V2) for publication in EMBO Molecular Medicine.

We have addressed all the issue that you have raised in your recent decision e-mail.

- we are displaying the full Western blots of Figure 2D.
- we have made statements about how many times each experiment was reproduced.
- we have added all p-values in the text.
- we have also prepared a PDF file containing the full Western blot for on-line display

I truly hope that the manuscript is now acceptable for publication.
