# Supplementary figures and images for: LPS resistance of SPRET/Ei mice is mediated by Gilz, encoded by the Tsc22d3 gene on the X chromosome
Source: EMBO Mol Med. 2013 Mar 5;5(3):456–70. doi: 10.1002/emmm.201201683 (PMC3598084; doi:10.1002/emmm.201201683)

Figure 2D

LIVER

LUNG

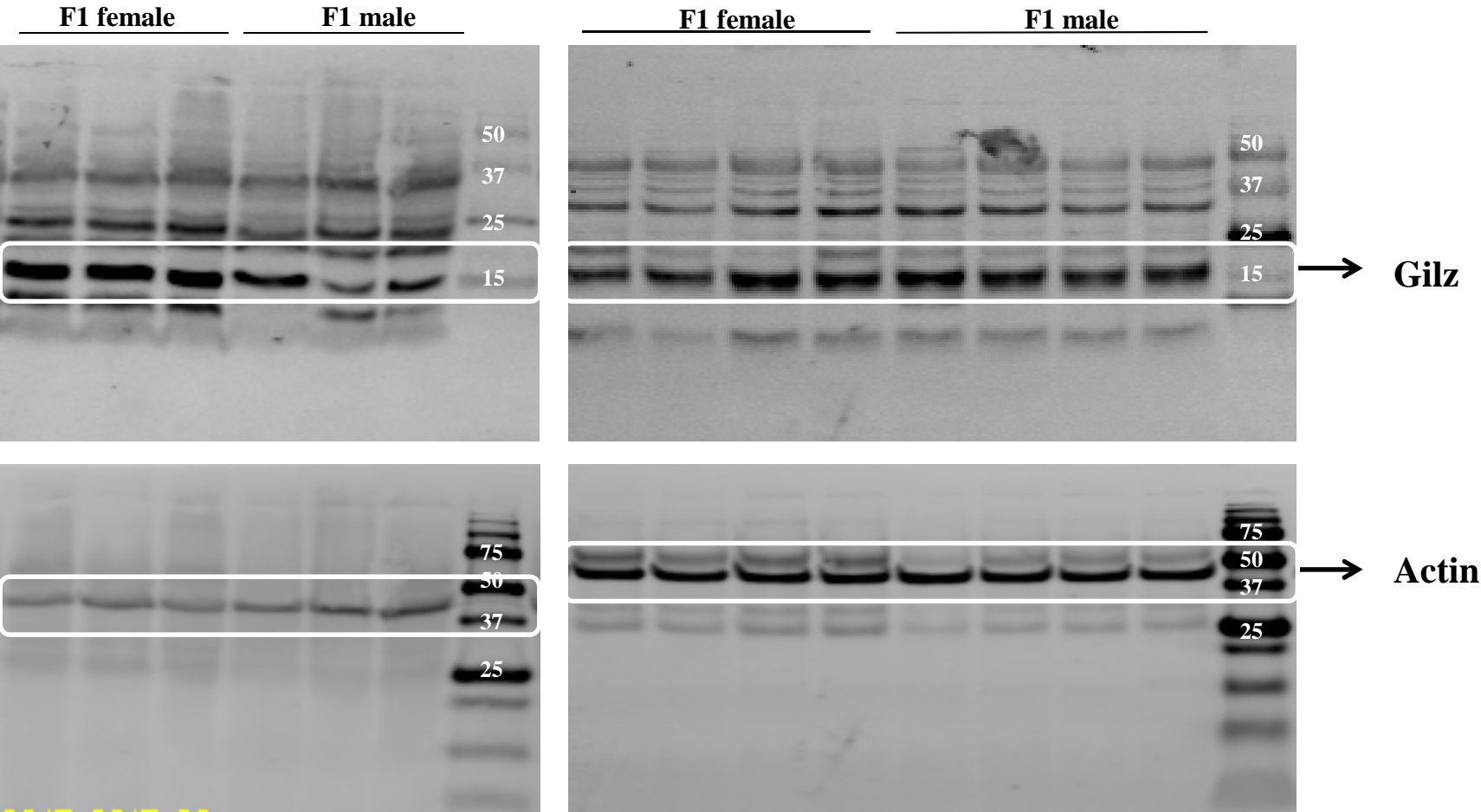

Supplement: Supplementary file 2 [file emmm0005-0456-SD2.pdf]
