# Supplementary material for: LPS resistance of SPRET/Ei mice is mediated by Gilz, encoded by the Tsc22d3 gene on the X chromosome
Source: EMBO Mol Med. 2013 Mar 5;5(3):456–70. doi: 10.1002/emmm.201201683 (PMC3598084; doi:10.1002/emmm.201201683)

Supporting Information

Tables

Supporting Information Table 1: List of GRE genes located in the X-located QTL region.

Figures

- Supporting Information Figure 1: The two QTL contributing to LPS resistance did not exhibit epistasis, suggesting that each QTL contributed additively to the LPS resistance.
- Supporting Information Figure 2: Sex hormones are not involved in the LPS resistance of (BxS)F1 females.
- Supporting Information Figure 3: Sequence variations located in the SPRET/Ei *Tsc22d3* sequence on the X chromosome.

Supporting Information Table 1: List of GRE genes located in the X-located QTL region. 72 annotated genes were identified in the 60-70 cM QTL region on the X chromosome (Fig. 1A, right graph), of which 41 genes contain putative GRE (1000 bp upstream of the transcription start site), found with the TRANSFAC matrix database 2011.3 (core matrix match threshold = 90%).

Gene start (bp)	Gene end (bp)	cM position	Gene name	Description	GRE number
134742704	134744119	60.2	Tex13a	testis expressed 13a	3
135448970	135545071	61.1	Nrk	Nik related kinase	3
135613796	135619775	61.2	Serpina7	serine peptidase inhibitor, member 7	8
135744581	135772872	61.3	Mum1l1	melanoma associated antigen 1-like 1	3
135868408	135872704	61.3	Trap1a	tumor rejection antigen P1A	3
136097855	136207684	61.4	Rnf128	ring finger protein 129	-
136219535	136287944	61.4	Tbc1d8b	TBC1 domain family, member 8B	-
136314191	136316898	61.4	Ripply1	Ripply1 homolog (zebrafish)	2
136335367	136345923	61.4	Cldn2	Claudin2	4
136356440	136406161	61.4	Morc4	Microrchidia 4	-
136477944	136533090	61.4	Rbm41	RNA binding motif protein 41	2
136542214	136597091	61.4	Nup62cl	Nucleoporin 62 C-terminal like	-
136916510	136968148	61.4	Frmpd3	FERM and PDZ domain containing 3	2
136991164	137010678	61.4	Prps1	Phosphoribosyl pyrophosphate synthetase1	1
137074076	137135061	61.4	Tsc22d3	TSC22 domain family, member 3	4
137199495	137302255	61.4	Mid2	Midline2	1
137342847	137347972	61.5	Tex13	testis expressed 13	-
137442147	137474011	61.6	Vsig1	V-set and immunoglobulin domain 1	2
137482968	137491267	61.7	Psmd10	Proteasome26S subunit, non-ATPase, 10	1
137491446	137598813	61.7	Atg4a	Autophagy-related 4A (yeast)	2
137599946	137908619	61.8	Col4a6	Collagen, type IV, alpha 6	-
137909928	138123777	62.2	Col4a5	Collagen, type IV, alpha 5	-
138145543	138159760	62.4	Irs4	Insulin receptor substrate 4	1
138515703	138631474	62.9	Gucy2f	Guanylate cyclase 2f	1
138661383	138674235	63.0	Nxt2	Nuclear transport factor2-like export factor	2
138740137	138740568	63.1	Kcne1l	Potassium voltage-gated channel, 1-like	-
138752536	138825078	63.1	Acsl4	Acyl-CoA synthetase long-chain 4	-
139115949	139278037	63.5	Tmem164	Transmembrain protein 164	-
139288017	139401271	63.7	Ammecr1	Alport syndrome, mental retardation, midface hypoplasia and elliptocytosis chromosomal region 1	-
139720217	139828727	64.2	Chrdl1	chordin-like 1	-
139953134	140232335	64.5	Pak3	p21 (CDKN1A)-activated kinase 3	1
140236785	140261957	64.8	Capn6	calpain 6	3
140290385	140367854	64.9	Dcx	doublecortin	3
140752564	140759725	65.4	Alg13	asparagine-linked glycosylation 13 homolog (S. cerevisiae)	-
140816214	141122723	65.5	Trpc5	transient receptor potential cation channel, subfamily C, member 5	-
141123450	141556954	65.8	Zcchc16	zinc finger, CCHC domain containing 16	-
141724904	141783437	66.5	Lhfpl1	lipoma HMGIC fusion partner-like 1	2

141880968	141939687	66.7	Amot	angiomotin	3
143397056	143631820	68.5	Htr2c	5-hydroxytryptamine receptor 2C	-
143818019	143863735	68.5	Il13ra2	interleukin 13 receptor, alpha 2	4
143887318	143888253	68.5	V1rb10	vomeronasal 1 receptor, B10	3
143906237	143988624	68.5	Lrch2	leucine-rich repeats and calponin homology domain containing 2	2
145292212	145358083	68.5	Luzp4	leucine zipper protein 4	1
145508825	145572695	68.5	Ott	ovary testis transcribed	6
146832316	146875680	68.5	Tmem29	transmembrane protein 29	-
146954062	147024387	68.5	Apex2	apurinic/apyrimidinic endonuclease 2	-
146981990	147005165	68.5	Alas2	aminolevulinic acid synthase 2, erythroid	3
147024464	147078403	68.5	Pfkfb1	6-phosphofructo-2-kinase/fructose-2,6-biphosphatase 1	-
147079847	147092126	68.5	Tro	trophinin	1
147240964	147248888	68.5	Maged2	melanoma antigen, family D, 2	3
147417684	147451865	68.5	Gnl3l	guanine nucleotide binding protein-like 3	3
147480693	147524064	68.5	Fgd1	FYVE, RhoGEF and PH domain 1	-
147521852	147531061	68.5	Tsr2	TSR2, 20S rRNA accumulation	3
147632683	147749671	68.5	Wnk3	WNK lysine deficient protein kinase 3	-
147955215	148065970	68.5	Phf8	PHD finger protein 8	-
148237870	148369960	68.5	Huwe1	HECT, UBA and WWE domain 1	-
148436444	148438983	68.5	Hsd17b10	hydroxysteroid (17-beta) dehydrogenase 10	-
148439125	148450838	68.5	Ribc1	RIB43A domain with coiled-coils 1	-
148450971	148496510	68.5	Smc1a	structural maintenance of chromosomes 1A	2
148578815	148659779	68.5	Iqsec2	IQ motif and Sec7 domain 2	2
148667563	148708317	68.5	Jarid1c	jumonji, AT rich interactive domain 1C	3
148771395	148777027	68.5	Tspyl2	TSPY-like 2	-
148779190	148802654	68.5	Gpr173	G-protein coupled receptor 173	1
149044052	149204008	68.5	Shroom2	shroom family member 2	-
149216529	149243189	68.5	Gpr143	G protein-coupled receptor 143	-
149441012	149443959	68.5	Usp51	ubiquitin specific protease 51	1
149470709	149472118	68.5	Mageh1	melanoma antigen, family H, 1	1
149553329	149567404	68.5	Foxr2	forkhead box R2	-
149574524	149606485	68.5	Rragb	Ras-related GTP binding B	-
149672588	149830677	68.5	Klf8	Kruppel-like factor 8	1
149932770	149936079	68.5	Ubqln2	ubiquilin 2	-
149993136	149993972	68.7	Cypt3	cysteine-rich perinuclear theca 3	3

Supporting Information Figure 1: The two QTL contributing to LPS resistance did not exhibit epistasis, suggesting that each QTL contributed additively to the LPS resistance. (A) Lack of epistasis between the two QTL contributing to LPS resistance was identified by the analysis of the interaction between the two QTL represented by the markers D2Mit510 and DXMit135, having the highest linkage score. The survival data was modeled as binomial data, followed by fitting a generalized linear model, incorporating a logit link function, as implemented in Genstat. The conclusion from the accumulated summary is there are significant QTL effects, but no interaction, indicative for the lack of epistasis. (B) QTL analysis of LPS resistance performed for each sex separately detected QTL on chromosomes 2 and X similar to those identified in the QTL analysis of (BxS)xB mice of both sexes combined. Linkage scores (expressed as $-\log_{10}(P)$) are shown according to genome position.

Supporting Information Figure 2: Sex hormones are not involved in the LPS resistance of (BxS)F1 females. (A-B) Ovariectomy does not influence the response of (BxS)F1 females to LPS. Ovariectomized (Ovx) (BxS)F1 females (n=5), sham operated (BxS)F1 females (n=5) and (BxS)F1 males (n=5) were i.p. injected with 500 µg LPS and body temperature was followed (A). Blood was collected 6 h after LPS challenge and serum IL6 levels were measured (B). Asterisks represent significant differences (Mann Whitney test) between each group of (BxS)F1 females and (BxS)F1 males. (C-D) Orchiectomy does not affect the response of (BxS)F1 males to LPS. (BxS)F1 females (n=5), castrated (BxS)F1 males (n=5) and sham operated (BxS)F1 males (n=5) were i.p. injected with 500 µg of LPS and body temperature was followed (C). Blood was collected 6 h after LPS challenge and serum IL6 levels were measured (D). Asterisks represent significant differences between each group of (BxS)F1 males and (BxS)F1 females. Alle experiments were performed once.

Supporting Information Figure 3: Sequence variations located in the SPRET/Ei *Tsc22d3* sequence on the X chromosome. The figure depicts the genomic structure of the canonical isoform of the *Tsc22d3* gene, carrying 3 different exons. Using the publicly available SPRET/Ei genome sequence of the Sanger Institute (http://www.sanger.ac.uk/), we identified many single nucleotide polymorphisms (SNPs) and few insertions and deletions (INDELS) between the SPRET/Ei and C57BL/6 *Tsc22d3* sequence.

Supporting Information Figure 1

A

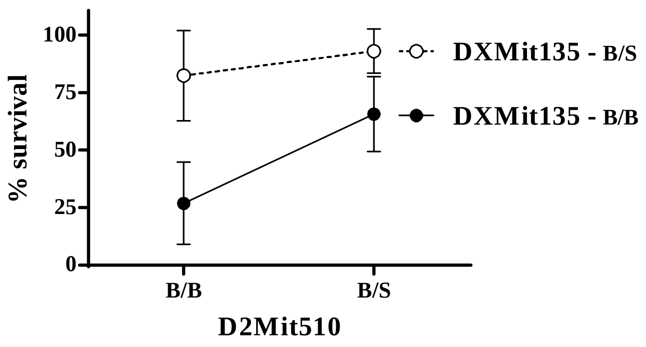

Change	d.f.	deviance	mean deviance	deviance ratio	approx chi prob
+ D2Mit510	1	9.843	9.843	9.84	0.002
+ DXMit135	1	20.868	20.868	20.87	<.001
+ D2Mit510.DXMit135	1	0.267	0.267	0.27	0.605
Residual	103	105.693	1.026		
Total	106	136.671	1.289		

B

Females

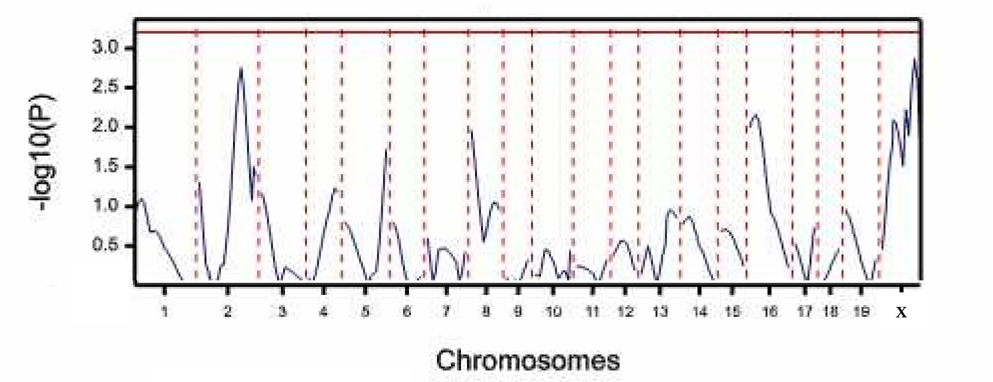

Males

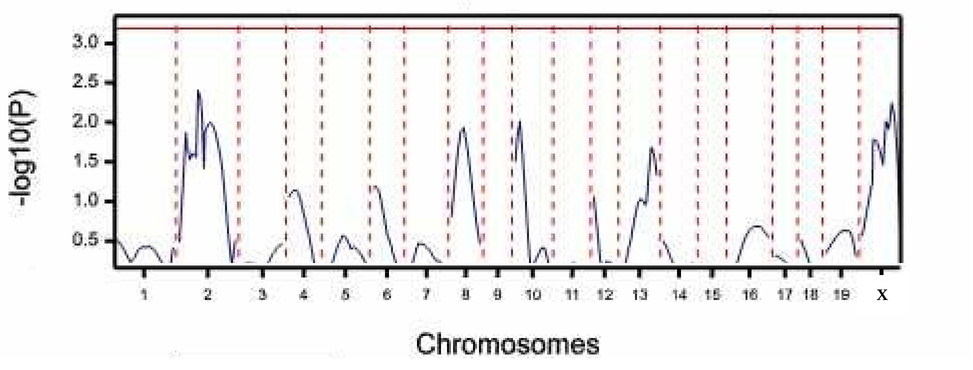

Supporting Information Figure 2

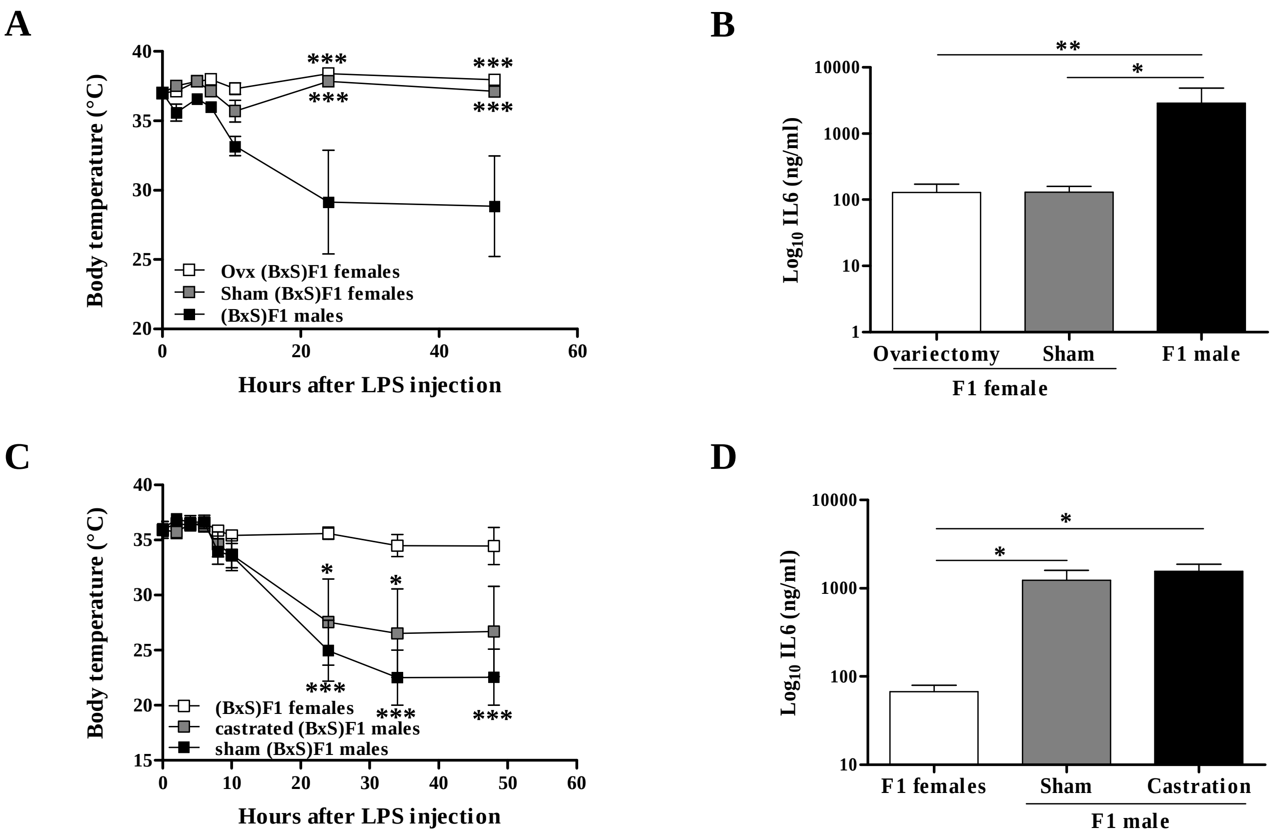

Supporting Information Figure 3

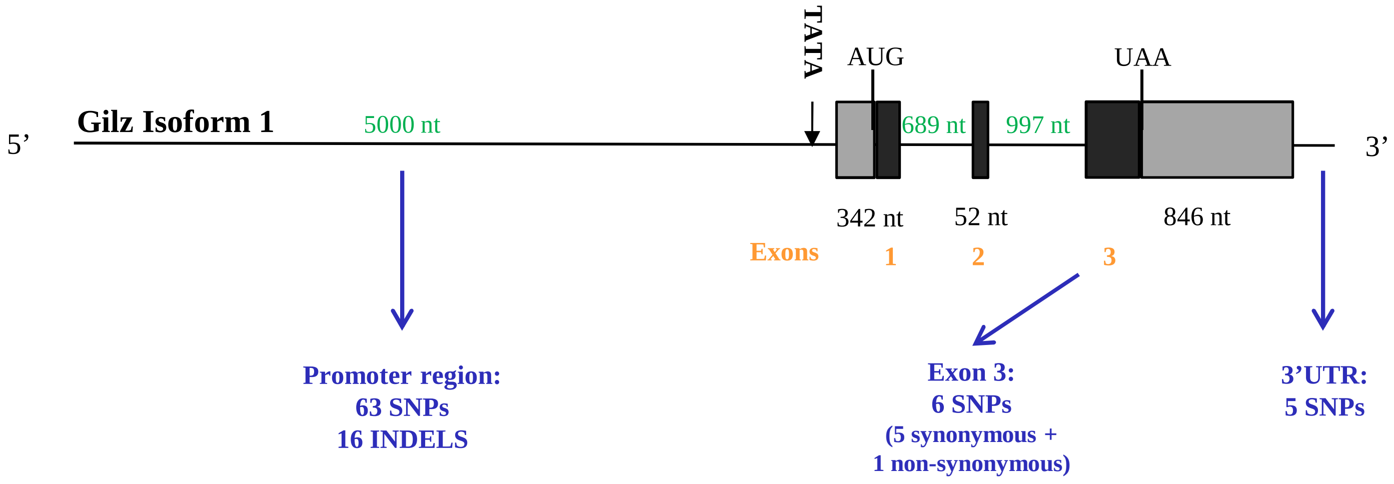

Supplement: Supplementary file 3 [file emmm0005-0456-SD3.pdf]
